# Parameter Identification for a Model of Neonatal Fc Receptor-Mediated Recycling of Endogenous Immunoglobulin G in Humans

**DOI:** 10.3389/fimmu.2019.00674

**Published:** 2019-04-08

**Authors:** Felicity Kendrick, Neil D. Evans, Oscar Berlanga, Stephen J. Harding, Michael J. Chappell

**Affiliations:** ^1^School of Engineering, University of Warwick, Coventry, United Kingdom; ^2^Department of Research and Development, The Binding Site Group Limited, Birmingham, United Kingdom

**Keywords:** biological systems, lumped-parameter systems, immunoglobulin G, neonatal Fc receptor, parameter estimation, structural identifiability

## Abstract

Salvage of endogenous immunoglobulin G (IgG) by the neonatal Fc receptor (FcRn) is implicated in many clinical areas, including therapeutic monoclonal antibody kinetics, patient monitoring in IgG multiple myeloma, and antibody-mediated transplant rejection. There is a clear clinical need for a fully parameterized model of FcRn-mediated recycling of endogenous IgG to allow for predictive modeling, with the potential for optimizing therapeutic regimens for better patient outcomes. In this paper we study a mechanism-based model incorporating nonlinear FcRn-IgG binding kinetics. The aim of this study is to determine whether parameter values can be estimated using the limited *in vivo* human data, available in the literature, from studies of the kinetics of radiolabeled IgG in humans. We derive mathematical descriptions of the experimental observations—timecourse data and fractional catabolic rate (FCR) data—based on the underlying physiological model. Structural identifiability analyses are performed to determine which, if any, of the parameters are unique with respect to the observations. Structurally identifiable parameters are then estimated from the data. It is found that parameter values estimated from timecourse data are not robust, suggesting that the model complexity is not supported by the available data. Based upon the structural identifiability analyses, a new expression for the FCR is derived. This expression is fitted to the FCR data to estimate unknown parameter values. Using these parameter estimates, the plasma IgG response is simulated under clinical conditions. Finally a suggestion is made for a reduced-order model based upon the newly derived expression for the FCR. The reduced-order model is used to predict the plasma IgG response, which is compared with the original four-compartment model, showing good agreement. This paper shows how techniques for compartmental model analysis—structural identifiability analysis, linearization, and reparameterization—can be used to ensure robust parameter identification.

## 1. Introduction

Immunoglobulin G (IgG) is the most abundant immunoglobulin (Ig) isotype in the circulation in humans, with a plasma concentration in healthy adults of 10–16 g l^−1^ (1). Its high concentration is facilitated by the neonatal Fc receptor (FcRn), which binds IgG in intracellular endosomes and transports it to the plasma membrane to be returned to the circulation. A proportion of IgG molecules that are not bound by FcRn are degraded in lysosomes. In this way, FcRn continually protects a proportion of the circulating IgG from degradation. The recycling mechanism is saturable, such that at high plasma IgG concentrations a greater proportion of plasma IgG is degraded. Conversely, at depleted plasma IgG concentrations, a greater proportion is recycled and the half-life is extended beyond the normal 23 days (2).

Recent publications have drawn attention to the importance of FcRn-mediated recycling of endogenous IgG in the bone marrow cancer multiple myeloma. In multiple myeloma, clonal plasma cells secrete an excess of monoclonal Ig into the circulation. Patients undergoing therapy are primarily monitored by quantification of Ig in blood serum samples (3). Mills et al. (4) have suggested that FcRn-mediated recycling of IgG may result in different response rates between patients with IgG-producing multiple myeloma and patients with IgA-producing multiple myeloma. Yan et al. (5) have also suggested that FcRn-mediated recycling of endogenous IgG in patients with multiple myeloma may shorten the half-life of the therapeutic monoclonal antibody daratumumab. These studies highlight the need for a parameterized model of endogenous IgG kinetics for investigating these clinical scenarios.

Numerous mathematical models of IgG kinetics have been presented in the literature, mostly with the aim of describing the pharmacokinetics of therapeutic monoclonal antibodies (mAbs) that are also regulated by FcRn. Many of these models are therefore pharmacokinetic in nature: their parameter values are obtained from animal experiments and they may be physiologically-based, with up to around 10 organs explicitly represented in the model (6–14). Pharmacokinetic models developed for specific mAbs may not be generalizable to endogenous IgG if, for example, they include details such as binding of the mAb to its target. In addition, mAb disposition may be adequately described by linear models in many cases where the plasma concentration of therapeutic mAb is substantially smaller than the plasma concentration of endogenous IgG and the latter is constant (13, 14). However, the assumption of a constant plasma concentration of IgG is not always appropriate; for example, in multiple myeloma the plasma IgG concentration typically shows large changes during the course of therapy. Relative to a less complex model, the more complex model will usually provide a better fit to observed data. However, this alone does not imply that all the parameters in the complex model can be estimated consistently, nor does it imply that the underlying assumptions of the complex model are valid (15).

In this paper we study a mechanism-based model with a single plasma compartment, rather than separate plasma compartments for different organs, which is accessible to measurement in humans. The model, which has been previously shown by Kim et al. (16) and Hattersley (17), has in total four compartments, representing IgG in plasma, IgG in a peripheral compartment (representing less rapidly perfused tissues), unbound IgG in intracellular endosomes and IgG bound to FcRn receptors in intracellular endosomes. The IgG-FcRn interaction is represented by nonlinear receptor-ligand binding kinetics (6, 7, 9). With reliable parameter values for humans, it may be possible to use this model to predict the responses of plasma IgG under various clinical conditions.

The aim of this study is to determine whether the model parameter values can be obtained using the limited *in vivo* human data that are available in the literature. The data are from studies of the kinetics of administered small doses of radiolabeled IgG when the subject's endogenous IgG is in steady state. We consider two measured outputs: the timecourse of the proportion of an administered dose of radiolabeled IgG remaining in plasma and in the body; and the relationship between the fractional catabolic rate and the quantity of endogenous IgG in plasma. Structural identifiability analysis is performed with respect to these outputs and structurally identifiable parameters are estimated from the data.

## 2. Mathematical Models and Data Description

### 2.1. The Four Compartment Model

**Figure 1:**
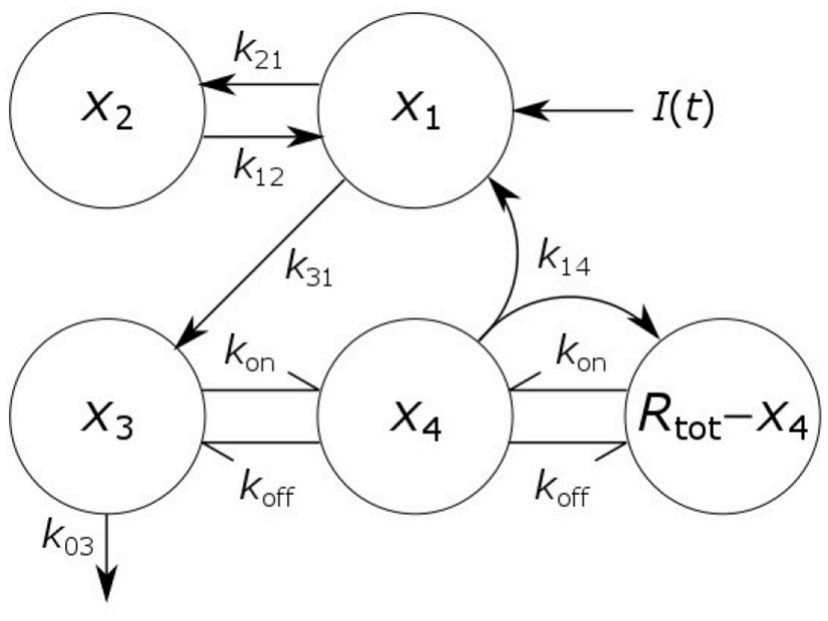
Schematic of four-compartment model of IgG metabolism. Arrows represent material flow between compartments and hooked arrows represent nonlinear receptor-ligand binding. The fifth compartment shown (*R*_tot_ − *x*_4_) represents unbound FcRn receptors and has been eliminated from the model equations. The arrow, labeled *k*_14_, from the IgG-FcRn complex compartment (*x*_4_) to the unbound FcRn receptor compartment (*R*_tot_ − *x*_4_), represents internalization of FcRn receptors from the plasma membrane to the endosome, after releasing IgG.

The model of IgG metabolism under study (16, 17) has four state variables, nine parameters, and an input function, *I*(*t*), representing the synthesis of IgG. The model equations are given by

(1) $$\begin{array}{l} \begin{array}{c} {ẋ}_{1}\left(t\right)=-\left(k_{21}+k_{31}\right)x_{1}\left(t\right)+k_{12}x_{2}\left(t\right)+k_{14}x_{4}\left(t\right)+I\left(t\right)\\ {ẋ}_{2}\left(t\right)=k_{21}x_{1}\left(t\right)-k_{12}x_{2}\left(t\right)\\ {ẋ}_{3}\left(t\right)=k_{31}x_{1}\left(t\right)-k_{03}x_{3}\left(t\right)-\frac{k_{\text{on}}}{v_{3}}x_{3}\left(t\right)\left(R_{\text{tot}}-x_{4}\left(t\right)\right)+k_{\text{off}}x_{4}\left(t\right)\\ {ẋ}_{4}\left(t\right)=\frac{k_{\text{on}}}{v_{3}}x_{3}\left(t\right)\left(R_{\text{tot}}-x_{4}\left(t\right)\right)-\left(k_{14}+k_{\text{off}}\right)x_{4}\left(t\right), \end{array} \end{array}$$

where *x*_1_(*t*), *x*_2_(*t*), *x*_3_(*t*), and *x*_4_(*t*) represent the quantities in μmol of IgG in plasma, IgG in a peripheral compartment, unbound IgG in endosomes and IgG bound to FcRn in endosomes, respectively. *I*(*t*) represents the rate of synthesis of IgG in µmol day^−1^. The rate constants, *k*_*ij*_, represent the rate of material flow from compartment *j* to compartment *i*, with the convention that 0 represents the environment outside the system. *k*_on_ and *k*_off_ are the receptor-ligand binding constants of IgG and FcRn. We denote the volumes of plasma, the peripheral compartment and the endosomes by *v*_1_, *v*_2_, and *v*_3_, respectively. We assume a constant total (bound and unbound) quantity of FcRn, *R*_tot_ (6). This means that the quantity of unbound FcRn is represented by [*R*_tot_ − *x*_4_(*t*)]. The state variables of the model and physiological interpretations of the parameters are summarized in Table 1. Note that all states and parameters can only take non-negative values. We refer to Figure 1 for a schematic of the model.

**Table 1:** States and parameters of four-compartment model of IgG metabolism, with parameter values sourced in the literature.

**Name**	**Units**	**Literature value**	**Physiological interpretation**
*x*_1_	μmol	–	Quantity of IgG in the central (plasma) compartment
*x*_2_	μmol	–	Quantity of IgG in the peripheral compartment
*x*_3_	μmol	–	Quantity of unbound IgG in intracellular endosomes
*x*_4_	μmol	–	Quantity of IgG-FcRn complexes in intracellular endosomes
*v*_1_	l	2.9^*^	Plasma volume
*v*_2_	l	–	Volume of peripheral compartment
*v*_3_	l	0.34^†^	Total volume of endosomes
*k*_21_	day^−1^	0.51^‡^	Rate constant of flow of IgG from plasma to peripheral compartment
*k*_31_	day^−1^	0.18^§^	Rate constant of flow of IgG from plasma into endosomes by pinocytosis
*k*_12_	day^−1^	0.41^‡^	Rate constant of flow of IgG from peripheral compartment to plasma
*k*_14_	day^−1^	5.0^¶^	Rate constant of flow of recycled IgG from endosomes back into plasma
*k*_03_	day^−1^	3.0^||^	Rate constant of degradation of unbound IgG in endosomes
*k*_on_	lμmol day^−1^	1,000^**^	Association rate constant of IgG-FcRn binding
*R*_tot_	μmol	14^¶^	Total quantity of FcRn receptors, bound and unbound
*k*_off_	day^−1^	100^**^	Dissociation rate constant of IgG-FcRn binding

* *Solomon et al. (18)*,

† *Shah and Betts (19)*,

‡ *Hattersley et al. (20)*,

§ *Waldmann and Strober (21)*,

¶ *Ferl et al. (6)*,

|| *Hansen and Balthasar (22)*,

** *Chen and Balthasar (10)*.

When the production rate of IgG is constant, *I*(*t*) = *I*_0_, the system has a stable equilibrium point given by

(2) $$\begin{array}{l} \begin{array}{c} {\hat{x}}_{1}=\frac{I_{0}\left(k_{03}k_{14}v_{3}+k_{03}k_{\text{off}}v_{3}+k_{\text{on}}I_{0}+k_{14}k_{\text{on}}R_{\text{tot}}\right)}{k_{31}\left(k_{03}v_{3}\left(k_{14}+k_{\text{off}}\right)+k_{\text{on}}I_{0}\right)}\\ {\hat{x}}_{2}=\frac{k_{21}}{k_{12}}{\hat{x}}_{1}\\ {\hat{x}}_{3}=\frac{I_{0}}{k_{03}}\\ {\hat{x}}_{4}=\frac{k_{\text{on}}I_{0}R_{\text{tot}}}{k_{03}v_{3}\left(k_{14}+k_{\text{off}}\right)+k_{\text{on}}I_{0}}. \end{array} \end{array}$$

A stability analysis for this equilibrium point is provided in the Supplementary Material.

### 2.2. *In vivo* Human Data From the Literature

The data available in the literature were obtained from tracer experiments. These studies entailed intravenous administration of a bolus dose of radiolabeled IgG (the tracer) and monitoring the proportion of the dose remaining in the blood and in the body over time. In this way the administered dose is distinguishable (by the experimenter) from the subject's own endogenous IgG. The quantity of administered tracer is small, so as not to perturb the steady state of the endogenous IgG. The purpose of tracer experiments is to enable observation of processes such as distribution and elimination undergone by the endogenous protein, whilst it is in steady state. The methods are described fully by Waldmann and Strober (21).

**Figure 2:**
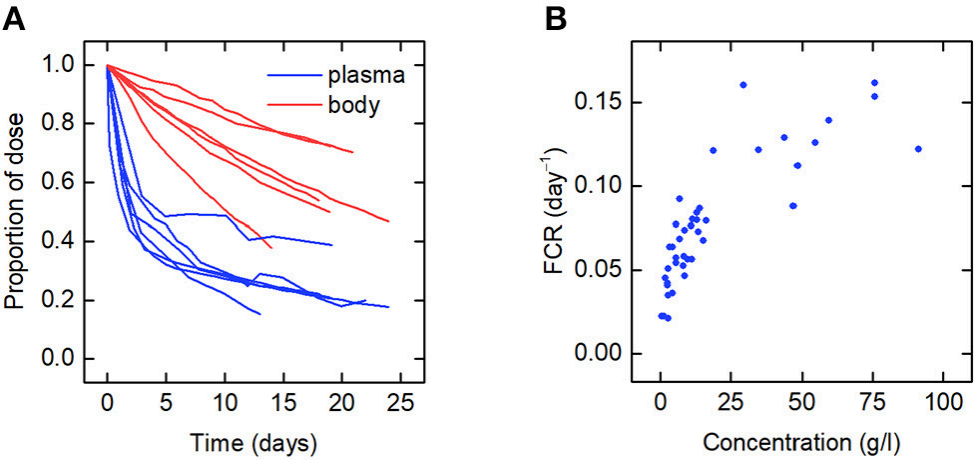
**(A)** Spaghetti plot of the proportion of administered IgG remaining in plasma (blue) and the body (red) in six subjects; data from Waldmann and Strober (21), Solomon et al. (18), and Waldmann and Terry (23). **(B)** Plasma concentration dependence of the fractional catabolic rate (FCR); redrawn from Waldmann and Strober (21), with permission from S. Karger AG, Basel.

The data for an individual subject consist of the timecourse of the proportion of the injected dose of IgG remaining in plasma and the timecourse of the proportion of dose remaining in the body. In this paper we use the data from six such plots available in the literature. We refer to the individuals as subjects A–F. The timecourse data for subjects A–D are from Solomon et al. (18), for subject E from Waldmann and Terry (23), and for subject F from Waldmann and Strober (21). Several of the individuals have health conditions which may result in an increased or decreased plasma IgG concentration. Subjects A and C have IgG multiple myeloma and subject D has macroglobulinemia. Subjects B, E, and F are referred to as “normal” subjects. A spaghetti plot of the data is shown in Figure 2A. Subjects A and D show slower dynamics and subject C shows faster dynamics. The dynamics of IgG in these subjects is assumed to be described by the same model, as given by Equations (1), however they may have had altered production rates of IgG due to the diseases.

Also available in the literature is a plot of the fractional catabolic rate (FCR) vs. the subject's plasma concentration of endogenous IgG, obtained from a group of individuals with a range of plasma IgG concentrations (21). The FCR is defined as the elimination rate of IgG as a fraction of the quantity of IgG in plasma. In practice the FCR is calculated from the rate at which the tracer dose leaves the body at time *t* divided by the proportion of tracer dose remaining in plasma at time *t*. The relationship between the FCR and the timecourse data is described further in section 2.6. A plot of the FCR vs. the plasma concentration of endogenous IgG for 41 individuals provided by Waldmann and Strober (21) is shown in Figure 2B. Each data point was derived from the timecourse data of an individual subject. All of the data described in this section were extracted from plots in the literature using the Digitizer tool in OriginPro 2016 (24).

### 2.3. Nonlinear Model of Coupled Tracer and Endogenous IgG Dynamics

The administered tracer and the endogenous IgG are assumed to be indistinguishable by the human body, that is they exhibit identical kinetic (input/output) behavior—a standard assumption in tracer studies (25). We therefore assume that the kinetics of both tracer and endogenous IgG are described by the model given by Equations (1). From Equations (1), letting *x*_*i*_(*t*) = *x*_*i*,T_(*t*) + *x*_*i*,E_(*t*), where *x*_*i*,T_(*t*) and *x*_*i*,E_(*t*) denote the quantities in μmol in compartment *i* of radiolabeled and endogenous IgG, respectively, gives

(3) $$\begin{array}{l} {\dot{x}}_{1,\text{T}}(t)=-(k_{21}+k_{31})x_{1,\text{T}}(t)+k_{12}x_{2,\text{T}}(t)+k_{14}x_{4,\text{T}}(t)\\ {\dot{x}}_{2,\text{T}}(t)=k_{21}x_{1,\text{T}}(t)-k_{12}x_{2,\text{T}}(t)\\ {\dot{x}}_{3,\text{T}}(t)=k_{31}x_{1,\text{T}}(t)-k_{03}x_{3,\text{T}}(t)-\frac{k_{\text{on}}}{v_{3}}x_{3,\text{T}}(t)(R_{\text{tot}}-x_{4,\text{E}}(t)\\ \;-x_{4,\text{T}}(t))+k_{\text{off}}x_{4,\text{T}}(t)\\ {\dot{x}}_{4,\text{T}}(t)=\frac{k_{\text{on}}}{v_{3}}x_{3,\text{T}}(t)\left(R_{\text{tot}}-x_{4,\text{E}}(t)-x_{4,\text{T}}(t)\right)-(k_{14}+k_{\text{off}})x_{4,\text{T}}(t)\\ {\dot{x}}_{1,\text{E}}(t)=-(k_{21}+k_{31})x_{1,\text{E}}(t)+k_{12}x_{2,\text{E}}(t)+k_{14}x_{4,\text{E}}(t)+I_{\text{E}}\\ {\dot{x}}_{2,\text{E}}(t)=k_{21}x_{1,\text{E}}(t)-k_{12}x_{2,\text{E}}(t)\\ {\dot{x}}_{3,\text{E}}(t)=k_{31}x_{1,\text{E}}(t)-k_{03}x_{3,\text{E}}(t)-\frac{k_{\text{on}}}{v_{3}}x_{3,\text{E}}(t)(R_{\text{tot}}-x_{4,\text{E}}(t)n\\ \;-x_{4,\text{T}}(t))+k_{\text{off}}x_{4,\text{E}}(t)\\ {\dot{x}}_{4,\text{E}}(t)=\frac{k_{\text{on}}}{v_{3}}x_{3,\text{E}}(t)\left(R_{\text{tot}}-x_{4,\text{E}}(t)-x_{4,\text{T}}(t)\right)\\ \;-(k_{14}+k_{\text{off}})x_{4,\text{E}}(t). \end{array}$$

*I*_E_ (μmol day^−1^) represents the production rate of endogenous IgG, which is assumed constant. All other parameters are defined in Table 1.

The dose of tracer administered at time *t* = 0 days is treated as a non-zero initial condition for *x*_1,T_(*t*). Tracer is administered to the plasma compartment only; therefore the initial conditions of the remaining tracer compartments are zero. The endogenous IgG is assumed to be in steady state throughout the experiment, such that the initial conditions of the endogenous IgG are given by the steady states in Equations (2), with *I*_0_ = *I*_E_. In summary, the initial conditions are given by

(4) $$\begin{array}{l} \begin{array}{c} x_{1,\text{T}}\left(0\right)=D\\ x_{2,\text{T}}\left(0\right)=x_{3,\text{T}}\left(0\right)=x_{4,\text{T}}\left(0\right)=0\\ x_{1,\text{E}}\left(0\right)={\hat{x}}_{1}\\ x_{2,\text{E}}\left(0\right)={\hat{x}}_{2}\\ x_{3,\text{E}}\left(0\right)={\hat{x}}_{3}\\ x_{4,\text{E}}\left(0\right)={\hat{x}}_{4}, \end{array} \end{array}$$

where ${\hat{x}}_{i}$ is the steady state quantity of endogenous IgG in compartment *i*, given by Equations (2), and *D* (μmol) is the administered dose of tracer.

The experimenter observes the proportion of the dose remaining in plasma [denoted by *y*_1_(*t*)] and in the body [denoted by *y*_2_(*t*)] during the experiment. The observation functions are thus given by

(5) $$\begin{array}{l} \begin{array}{c} y_{1}\left(t\right)=\frac{x_{1,\text{T}}\left(t\right)}{D}\\ y_{2}\left(t\right)=\frac{x_{1,\text{T}}\left(t\right)+x_{2,\text{T}}\left(t\right)+x_{3,\text{T}}\left(t\right)+x_{4,\text{T}}\left(t\right)}{D}. \end{array} \end{array}$$

### 2.4. Linearized Model of Tracer Dynamics

Provided that the administered dose of tracer is sufficiently small, the tracer kinetics can be approximated using the Taylor series expansion of the model state about the equilibrium point. In this way a linear model of the experiment, valid in a neighborhood of the equilibrium point, is derived. Our derivation is provided in the Supplementary Material. The derivation of a linearized model for tracer dynamics from a general compartmental model is provided by Anderson (26).

The linear equations describing the tracer kinetics are given by

(6) $$\begin{array}{l} \begin{array}{c} {ẋ}_{1,\text{T}}\left(t\right)=-\left(k_{21}+k_{31}\right)x_{1,\text{T}}\left(t\right)+k_{12}x_{2,\text{T}}\left(t\right)+k_{14}x_{4,\text{T}}\left(t\right)\\ {ẋ}_{2,\text{T}}\left(t\right)=k_{21}x_{1,\text{T}}\left(t\right)-k_{12}x_{2,\text{T}}\left(t\right)\\ {ẋ}_{3,\text{T}}\left(t\right)=k_{31}x_{1,\text{T}}\left(t\right)-k_{03}x_{3,\text{T}}\left(t\right)-k_{43}x_{3,\text{T}}\left(t\right)+k_{34}x_{4,\text{T}}\left(t\right)\\ {ẋ}_{4,\text{T}}\left(t\right)=k_{43}x_{3,\text{T}}\left(t\right)-\left(k_{14}+k_{34}\right)x_{4,\text{T}}\left(t\right) \end{array} \end{array}$$

where *x*_1,T_(*t*), *x*_2,T_(*t*), *x*_3,T_(*t*), and *x*_4,T_(*t*) represent the quantities of radiolabeled IgG in the central compartment, in the peripheral compartment, unbound in intracellular endosomes, and bound to FcRn in intracellular endosomes, respectively. The new parameters *k*_34_ and *k*_43_ are given by

(7) $$\begin{array}{l} \begin{array}{c} k_{34}=k_{\text{off}}\\ k_{43}=\frac{k_{\text{on}}\left(R_{\text{tot}}-{\hat{x}}_{4}\right)}{v_{3}}=\frac{k_{\text{on}}R_{\text{tot}}k_{03}\left(k_{14}+k_{\text{off}}\right)}{I_{\text{E}}k_{\text{on}}+k_{03}v_{3}\left(k_{14}+k_{\text{off}}\right)}. \end{array} \end{array}$$

All other parameters are defined in Table 1. The initial conditions are given by the first two equations of Equations (4) and the observation functions are given by Equations (5).

### 2.5. Comparison of Nonlinear Model and Linearized Model for Large Tracer Doses

**Figure 3:**
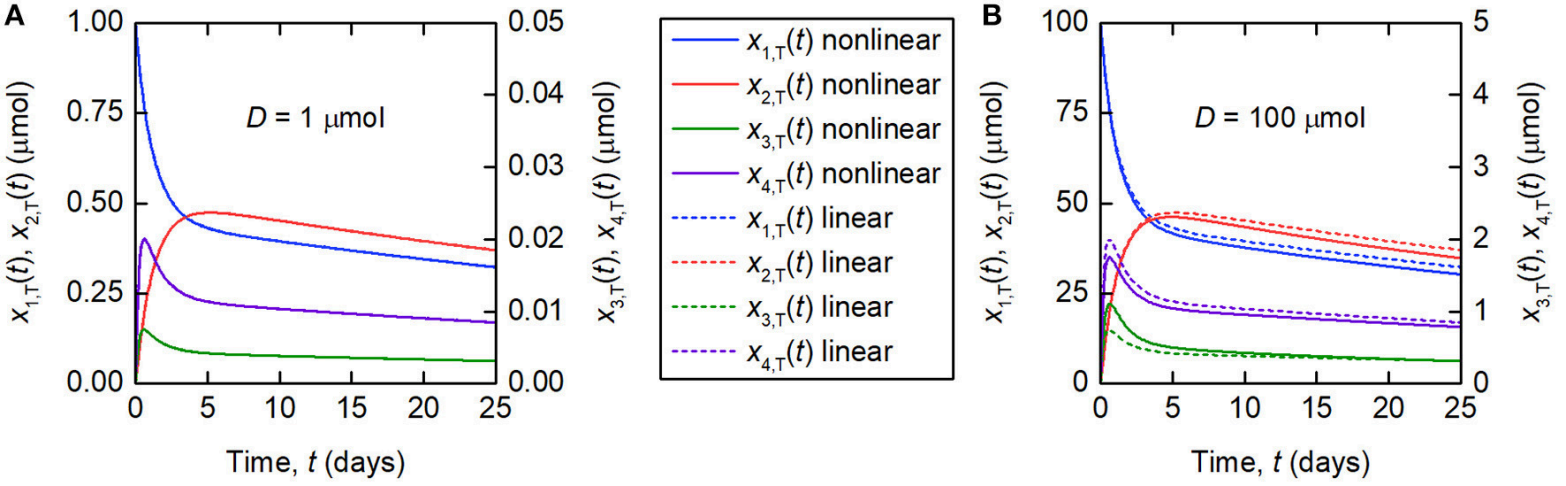
Simulations of the quantities of tracer in each compartment after administration at *t* = 0 days, for a tracer dose of **(A)** 1 μmol and **(B)** 100 μmol. The nonlinear model (Equations 3–4) is represented by solid lines and the linearized model (Equations 6) by dashed lines. The linearized model is valid for the smaller dose but not for the larger dose. Note the different scales for *x*_1,T_(*t*) and *x*_2,T_(*t*), and *x*_3,T_(*t*) and *x*_4,T_(*t*), respectively.

The linearization of the model of timecourse observations relies on the assumption of a sufficiently small dose of tracer, such that the endogenous IgG can be assumed to remain in steady state. A typical tracer dose is between 3 · 10^−3^ and 7 · 10^−3^ μmol (18). Simulations of the quantity of tracer in each compartment are shown in Figure 3. In Figure 3A, a dose of *D* = 1 µmol is assumed and in Figure 3B, a dose of *D* = 100 µmol is assumed. The value of 1 μmol was chosen to show that the linear model is a valid approximation of the nonlinear model, even when the dose is more than 100 times typical tracer doses. The extremely large value of 100 μmol was chosen specifically to show the dynamics of the linearized model when it is not a valid approximation of the nonlinear model. The parameter values in Table 1 are used. A normal IgG synthesis rate of *I*_E_ = 15 µmol day^−1^ was used; however the linearized model was still valid for *D* = 1 µmol when comparatively very small values of *I*_E_ were used. We find that, for a dose of 1 μmol and the particular parameter values used, the linearized model is a valid approximation of the full nonlinear model over a 25-day simulated time course. When the dose is increased to 100 μmol, the assumption that the steady state is not perturbed by the administered dose no longer holds and the two models give different simulation results for the quantities of tracer.

### 2.6. Fractional Catabolic Rate

We recall that the FCR (μmol day^−1^) is defined as the elimination rate of IgG as a fraction of the quantity of IgG in plasma and can be defined with respect to the tracer or with respect to the endogenous IgG. The FCR with respect to the tracer is therefore given by

(8) $$\begin{array}{l} {\text{FCR}}_{\text{T}}\left(t\right)=\frac{k_{03}x_{3,\text{T}}\left(t\right)}{x_{1,\text{T}}\left(t\right)}, \end{array}$$

where *x*_3,T_(*t*) and *x*_1,T_(*t*) are given by the solution of Equations (6).

**Figure 4:**
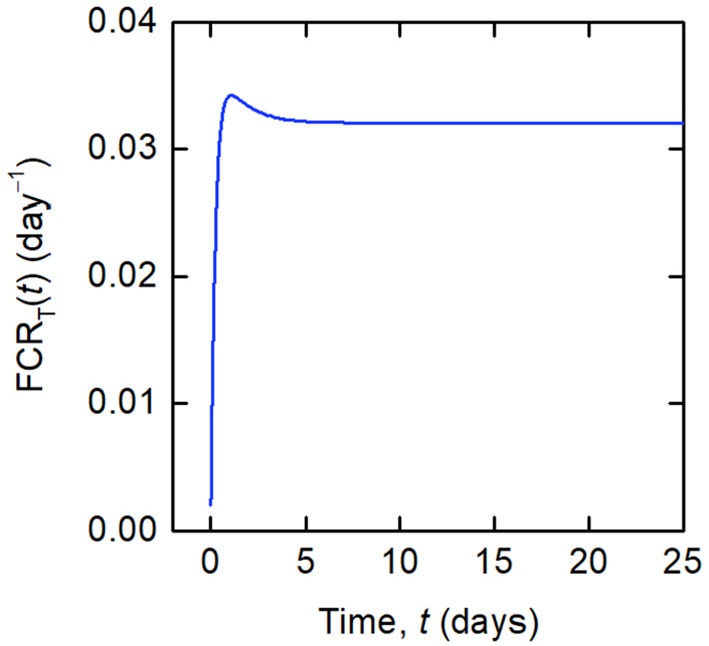
Simulation of FCR_T_(*t*) given by Equations (12) and (8), for the parameter values in Table 1 and dose *D* = 0.01 μmol.

Whilst a single value of the FCR is measured for an individual subject (see Figure 2B), in actuality FCR_T_(*t*) is not constant, as shown by the dependence on time in Equation (8). A simulation of FCR_T_(*t*) during the experiment is shown in Figure 4. After around day 5, for the particular parameter values used, FCR_T_(*t*) approaches a steady state value, which is denoted here by FCR_T,∞_:

(9) $$\begin{array}{l} {\text{FCR}}_{\text{T},\infty }=\underset{t\to \infty }{\lim}\frac{k_{03}x_{3,\text{T}}\left(t\right)}{x_{1,\text{T}}\left(t\right)}. \end{array}$$

Solving Equations (6) gives

(10) $$\begin{array}{l} x_{i,\text{T}}\left(t\right)=A_{i1}\exp\left(\lambda_{1}t\right)+A_{i2}\exp\left(\lambda_{2}t\right)+A_{i3}\exp\left(\lambda_{3}t\right)\\ +A_{i4}\exp\left(\lambda_{4}t\right),i=1,\ldots ,4, \end{array}$$

where *A*_*ij*_ and λ_*j*_ (*j* = 1, …, 4) are expressions in terms of the model parameters and |λ_1_| > |λ_2_| > |λ_3_| > |λ_4_|. After sufficient time, *x*_*i*, T_(*t*) can be approximated by *A*_*i*4_ exp(λ_4_*t*); thus, FCR_T,∞_ is given by

(11) $$\begin{array}{l} {\text{FCR}}_{\text{T},\infty }=k_{03}\frac{A_{34}\exp\left(\lambda_{4}t\right)}{A_{14}\exp\left(\lambda_{4}t\right)}=k_{03}\frac{A_{34}}{A_{14}}. \end{array}$$

The expressions for *A*_34_ and *A*_14_ in terms of the model parameters are extremely long. The Mathematica (27) code for generating the expressions for *A*_*ij*_ and λ_*j*_ is provided in the Supplementary Material.

Noting that there is only elimination from the system and no input for *t* > 0, FCR_T_(*t*) is equal to the rate of change of radiolabeled IgG in all compartments, divided by the quantity of radiolabeled IgG in plasma:

(12) $$\begin{array}{l} {\text{FCR}}_{\text{T}}\left(t\right)=\frac{-\left({ẋ}_{1,\text{T}}\left(t\right)+{ẋ}_{2,\text{T}}\left(t\right)+{ẋ}_{3,\text{T}}\left(t\right)+{ẋ}_{4,\text{T}}\left(t\right)\right)}{x_{1,\text{T}}\left(t\right)}=\frac{-{ẏ}_{2}\left(t\right)}{y_{1}\left(t\right)}. \end{array}$$

From Equation (12), it can be seen that FCR_T_(*t*) is equal to the slope of the observation *y*_2_(*t*) divided by *y*_1_(*t*), showing how FCR_T_(*t*) can be obtained from the observations *y*_1_(*t*) and *y*_2_(*t*). In practice, the experimenter obtains a value for FCR_T_(*t*_*N*_), where *t*_*N*_ is a time toward the end of the experiment, such that FCR_T_(*t*_*N*_) can be assumed a close approximation of FCR_T,∞_. Henceforth, the quantity obtained from experiments, FCR_T_(*t*_*N*_), is referred to simply as FCR_T_.

It is also possible to derive an expression for the FCR with respect to the endogenous IgG, FCR_E_. If the endogenous IgG is assumed to remain in steady state, then from the definition of the FCR,

(13) $$\begin{array}{l} {\text{FCR}}_{\text{E}}=\frac{k_{03}{\hat{x}}_{3}}{{\hat{x}}_{1}}, \end{array}$$

where ${\hat{x}}_{1}$ and ${\hat{x}}_{3}$ are the quantities of IgG in compartments 1 and 3 in steady state, given by Equations (2). Substituting the expression for ${\hat{x}}_{3}$ from Equations (2) into Equation (13), eliminating *I*_0_ in favor of ${\hat{x}}_{1}$ using the first equation of Equations (2), and setting ${\hat{x}}_{1}=x_{1,\text{E}}$, gives the following expression for the FCR_E_ in terms of the quantity of IgG in plasma, *x*_1,E_:

(14) $$\begin{array}{l} {\text{FCR}}_{\text{E}}=\frac{1}{2k_{\text{on}}x_{1,\text{E}}}(k_{31}k_{\text{on}}x_{1,\text{E}}-k_{14}k_{\text{on}}R_{\text{tot}}-k_{03}k_{14}v_{3}\\ \;-k_{03}k_{\text{off}}v_{3}+\{4k_{03}k_{31}(k_{14}+k_{\text{off}})k_{\text{on}}x_{1,\text{E}}v_{3}\\ \;+(-k_{31}k_{\text{on}}x_{1,\text{E}}+k_{14}k_{\text{on}}R_{\text{tot}}+k_{03}k_{14}v_{3}\\ \;+{{k_{03}k_{\text{off}}v_{3})}^{2}\}}^{1/2}). \end{array}$$

## 3. Results

### 3.1. Parameter Identification Using Tracer Timecourse Data

In this section we investigate whether it is possible to estimate unknown model parameter values by fitting the linear approximation described in section 2.4 to the timecourse data described in section 2.2. Firstly, a structural identifiability analysis is performed. Parameter values are then estimated from the data by fitting the linearized model described by Equations (6) to the data.

#### 3.1.1. Structural Identifiability Analysis

Structural identifiability addresses the question of whether model parameters can be uniquely identified from the available observations, under the assumption of the availability of ideal (i.e. noise-free) and continuous observational data. Here we determine which of the model parameters are structurally uniquely identifiable from the observations *y*_1_(*t*) and *y*_2_(*t*), given by Equations (4–6). The unknown parameter vector is given by $\theta ={\left(k_{21},k_{31},k_{12},k_{14},k_{03},k_{43},k_{34}\right)}^{\text{T}}$.

The transfer function method is used (28). To apply this approach the system described by Equations (4–6) is re-written in vector-matrix notation as

(15) $$\begin{array}{l} \begin{array}{c} {\dot{\text{x}}}_{\text{T}}\left(t,\theta \right)=\text{A}\left(\theta \right){\text{x}}_{\text{T}}\left(t\right)+\text{B}\left(\theta \right)u\left(t\right)\\ {\text{x}}_{\text{T}}\left(0,\theta \right)=0\\ \text{y}\left(t,\theta \right)=\text{C}\left(\theta \right){\text{x}}_{\text{T}}\left(t\right), \end{array} \end{array}$$

where ${\text{x}}_{\text{T}}\left(t,\theta \right)={\left(x_{1,\text{T}}\left(t\right),x_{2,\text{T}}\left(t\right),x_{3,\text{T}}\left(t\right),x_{4,\text{T}}\left(t\right)\right)}^{\text{T}}$ and $\text{y}\left(t,\theta \right)={\left(y_{1}\left(t\right),y_{2}\left(t\right)\right)}^{\text{T}}$ are column vectors representing the state vector and the observation vector, respectively, and *u*(*t*) represents the single input to the system, an impulse at time *t* = 0, given by *u*(*t*) = δ(*t*). ***A**(**θ**)* is a 4 × 4 matrix, ***B**(**θ**)* is a column vector and ***C**(**θ**)* is a 2 × 4 matrix. ***A**(**θ**)*, ***B**(**θ**)*, and ***C**(**θ**)* are given by

(16) $$\begin{array}{l} \begin{array}{c} \text{A}\left(\theta \right)=\left(\begin{array}{cccc} -\left(k_{21}+k_{31}\right) & k_{12} & 0 & k_{14}\\ k_{21} & -k_{12} & 0 & 0\\ k_{31} & 0 & -\left(k_{03}+k_{43}\right) & k_{34}\\ 0 & 0 & k_{43} & -\left(k_{14}+k_{34}\right) \end{array}\right),\\ \text{B}\left(\theta \right)=\left(\begin{array}{c} D\\ 0\\ 0\\ 0 \end{array}\right),\\ \text{C}\left(\theta \right)=\left(\begin{array}{cccc} \frac{1}{D} & 0 & 0 & 0\\ \frac{1}{D} & \frac{1}{D} & \frac{1}{D} & \frac{1}{D} \end{array}\right). \end{array} \end{array}$$

Note that the administration of a bolus dose of size *D* is now represented as an impulse at time *t* = 0, rather than a non-zero initial condition, such that ${\text{x}}_{\text{T}}\left(0,\theta \right)={\left(0,0,0,0\right)}^{\text{T}}$.

Taking Laplace transforms of Equations (15), the input-output relation is given by ***Y***(*s*) = ***G***(*s*)*U*(*s*), where ***G***(*s*) is the transfer function matrix, given by ***G***(*s*) = ***C***(**θ**)(*s**I*** − ***A***(**θ**))^−1^***B***(**θ**), where ***I*** is the 4 × 4 identity matrix. **G**(*s*) has two elements, corresponding to the two observed outputs, which are given by

(17) $$\begin{array}{l} \begin{array}{c} G_{1}\left(s\right)=\frac{\phi_{1}+\phi_{2}s+\phi_{3}s^{2}+s^{3}}{\phi_{4}+\phi_{5}s+\phi_{6}s^{2}+\phi_{7}s^{3}+s^{4}}\\ G_{2}\left(s\right)=\frac{\phi_{8}+\phi_{9}s+\phi_{10}s^{2}+s^{3}}{\phi_{11}+\phi_{12}s+\phi_{13}s^{2}+\phi_{14}s^{3}+s^{4}}, \end{array} \end{array}$$

where the coefficients of *s*, $\Phi \left(\theta \right)={\left(\phi_{1}\left(\theta \right),\phi_{2}\left(\theta \right),\ldots ,\phi_{14}\left(\theta \right)\right)}^{\text{T}}$, are nonlinear expressions in the parameters. The coefficients of *s*, **Φ**(**θ**), are given by

(18) $$\begin{array}{l} \phi_{1}(\theta )=k_{12}\left(k_{03}\left(k_{14}+k_{34}\right)+k_{14}k_{43}\right)\\ \phi_{2}(\theta )=k_{03}\left(k_{12}+k_{14}+k_{34}\right)+k_{14}k_{43}+k_{12}\left(k_{14}+k_{34}+k_{43}\right)\\ \phi_{3}(\theta )=k_{03}+k_{12}+k_{14}+k_{34}+k_{43}\\ \phi_{4}(\theta )=\phi_{11}(\theta )=k_{03}k_{12}k_{31}(k_{14}+k_{34})\\ \phi_{5}(\theta )=\phi_{12}(\theta )=k_{03}((k_{21}+k_{31})(k_{14}+k_{34})+k_{12}(k_{14}+k_{31}\\ \;+k_{34}))+k_{14}k_{21}k_{43}+k_{12}(k_{14}(k_{31}+k_{43})+k_{31}(k_{34}+k_{43}))\\ \phi_{6}(\theta )=\phi_{13}(\theta )=k_{14}k_{21}+k_{14}k_{31}+k_{21}k_{34}+k_{31}k_{34}+k_{03}(k_{12}\\ \;+k_{14}+k_{21}+k_{31}+k_{34})+k_{14}k_{43}+k_{21}k_{43}+k_{31}k_{43}+k_{12}\\ \left(k_{14}+k_{31}+k_{34}+k_{43}\right)\\ \phi_{7}(\theta )=\phi_{10}(\theta )=\phi_{14}(\theta )=k_{03}+k_{12}+k_{14}+k_{21}+k_{31}\\ \;+k_{34}+k_{43}\\ \phi_{8}(\theta )=k_{03}(k_{12}+k_{21})(k_{14}+k_{34})+k_{14}k_{21}k_{43}\\ \;+k_{12}(k_{14}(k_{31}+k_{43})+k_{31}(k_{34}+k_{43}))\\ \phi_{9}(\theta )=k_{14}k_{21}+k_{14}k_{31}+k_{21}k_{34}+k_{31}k_{34}+k_{03}(k_{12}+k_{14}\\ +k_{21}+k_{34})+k_{14}k_{43}+k_{21}k_{43}+k_{31}k_{43}\\ +k_{12}(k_{14}+k_{31}+k_{34}+k_{43}). \end{array}$$

The coefficients **Φ**(**θ**) are unique with respect to the input-output relationship represented by the transfer function. Introducing an alternative parameter vector, $\bar{\theta }={\left({\bar{k}}_{21},{\bar{k}}_{31},{\bar{k}}_{12},{\bar{k}}_{14},{\bar{k}}_{03},{\bar{k}}_{43},{\bar{k}}_{34}\right)}^{\text{T}}$, and equating $\Phi \left(\theta \right)=\Phi \left(\bar{\theta }\right)$, the resulting set of simultaneous equations is solved for **θ** using the Solve function in Mathematica (27). The only solution is $\theta =\bar{\theta }$; therefore all of the parameters in **θ** are structurally uniquely identifiable.

#### 3.1.2. Parameter Estimation

The parameter vector $\theta ={\left(k_{21},k_{31},k_{12},k_{14},k_{03},k_{43},k_{34}\right)}^{\text{T}}$ was estimated for each subject using unweighted least squares, by fitting the timecourse data described in section 2.2. The “true” parameter vector for an individual is denoted by **θ**_0_. For an individual subject it is assumed that *y*_*i*_(*t*, **θ_0_**), *i* = 1, 2, is observed with error at measurement times $t_{1}^{\left(i\right)},\ldots ,t_{N_{i}}^{\left(i\right)},i=1,2$, where $t_{1}^{\left(1\right)}=t_{1}^{\left(2\right)}=0$. The observed (with error) values of *y*_*i*_(*t*, **θ_0_**), *i* = 1, 2, are now denoted by ${\tilde{y}}_{i}(t_{j}^{\left(i\right)},\theta_{0})$ for *i* = 1, 2 and *j* = 1, …, *N*_*i*_. Both outputs *y*_1_ and *y*_2_ were fitted simultaneously, therefore the cost functional for **θ** is given by

(19) $$\begin{array}{l} J\left(\theta_{0},\theta \right)=\overset{2}{\underset{i=1}{\sum }}J_{i}\left(\theta_{0},\theta \right), \end{array}$$

where

(20) $$\begin{array}{l} J_{i}(\theta_{0},\theta )=\sum_{j=1}^{N_{i}}{\left({\tilde{y}}_{i}(t_{j}^{\left(i\right)},\theta_{0})-y_{i}(t_{j}^{\left(i\right)},\theta )\right)}^{2}. \end{array}$$

Differential evolution was implemented using the NonlinearModelFit function in Mathematica (27). The differential evolution algorithm was chosen because there is little information available about the parameters, in particular the parameters *k*_14_, *k*_03_, *k*_43_, and *k*_34_. Differential evolution is a stochastic, global minimization algorithm that does not require the user to specify initial guesses for the parameter values (29). All parameters were constrained to be positive. The maximum number of iterations was set to 5,000, which was sufficient for the algorithm to converge in all cases. In differential evolution an initial population of parameter vectors is generated randomly. The algorithm was run for each subject's data with integer seeds for the pseudorandom number generator between 1 and 10; thus 10 estimates for **θ** were obtained for each subject.

Differential evolution maintains a population of parameter vectors which evolves iteratively. For each new generation of the algorithm, a mutant and trial vector are produced from the current generation and the trial vector is compared with a target vector from the current generation. Either the target or trial vector is selected to move forward to the new generation based on which has the smallest value of the cost function to be minimized. The scaling factor (*SF*) is used to produce the mutant vector and generally a larger value of *SF* means a broader search of the parameter space. The crossover probability (*CR*) is the probability that each element of the mutant vector is used to produce the trial vector, rather than the corresponding element of the target vector. *SF* and *CR* were tuned by trial and error for each subject. The settings *F* = 0.5 and *CR* = 0.9 were tried initially, as recommended by Storn and Price (29) for faster convergence. For subjects C and F the settings were adjusted to *F* = 0.7, for a broader search of the parameter space, and *CR* = 0.95, to speed convergence. The settings for the differential evolution algorithm are given in Table 2.

**Table 2:** Settings for differential evolution.

	**Subject**					
	**A**	**B**	**C**	**D**	**E**	**F**
Scaling factor (**SF**)	0.5	0.5	0.7	0.5	0.5	0.7
Crossover probability (**CR**)	0.9	0.9	0.95	0.9	0.9	0.95

Each run of the algorithm, with a unique seed for the pseudorandom number generator, can produce unique parameter estimates; it is therefore recommended to perform multiple runs with unique, randomly chosen starting populations of parameter vectors (29). The parameter estimates and root mean square error (RMSE) for each run and each subject are tabulated in Table 3. The parameter estimates from multiple runs should be close to one another so that they can be averaged (29, 30); however, in some cases, the different runs give very different parameter estimates, implying that the algorithm has difficulty finding the global minimum and that there may be many local minima. It is therefore not certain that the global minimum has been found for each subject. It is also possible that certain parameters are highly correlated, such that different parameter vectors produce very similar model outputs. This is reflected in the diversity of parameter vectors obtained within subjects using differential evolution.

**Table 3:** Parameter values estimated from timecourse data.

	**Run**	**Parameter (all have units day^−1^)**							**RMSE**
		***k*_21_**	***k*_31_**	***k*_12_**	***k*_14_**	***k*_03_**	***k*_43_**	***k*_34_**	
Subject A	1	0.391	0.158	1.29	0.0628	0.261	1.88	0.206	0.0124
	2	0.391	0.159	1.29	0.0623	0.294	2.23	0.216	0.0124
	3	0.390	0.159	1.29	0.0616	0.341	2.70	0.225	0.0124
	4	0.390	0.159	1.29	0.0612	0.363	2.91	0.227	0.0124
	5	0.388	0.139	1.11	0.0699	0.0881	0.209	0.0279	0.0123
	6	0.391	0.160	1.30	0.0611	0.395	3.25	0.233	0.0124
	7	0.392	0.160	1.30	0.0616	0.365	2.95	0.229	0.0124
	8	0.386	0.159	1.27	0.0615	0.327	2.54	0.221	0.0124
	9	0.391	0.159	1.29	0.0617	0.336	2.64	0.224	0.0124
	10	0.390	0.159	1.29	0.0619	0.307	2.35	0.218	0.0124
Subject B	1	1.72	0.174	2.96	0.151	1.04	1.23	1.09 · 10^−16^	0.00858
	2	0.101	0.732	0.147	3.18	0.208	1.72	0.00	0.00859
	3	0.0986	1.09	0.146	2.49	0.408	20.2	7.16	0.00865
	4	1.73	0.174	2.98	0.151	1.04	1.23	0.00	0.00858
	5	1.72	0.174	2.96	0.151	1.04	1.23	0.00	0.00858
	6	1.72	0.174	2.96	0.151	1.04	1.23	0.00	0.00858
	7	1.72	0.174	2.96	0.151	1.04	1.23	0.00	0.00858
	8	1.72	0.174	2.96	0.151	1.04	1.23	3.69 · 10^−15^	0.00858
	9	0.101	0.732	0.147	3.18	0.208	1.72	0.00	0.00859
	10	1.72	0.174	2.96	0.151	1.04	1.23	0.00	0.00858
Subject C	1	0.0217	0.438	6.47 · 10^−16^	0.527	0.580	2.10	0.332	0.00682
	2	0.346	0.160	0.537	0.00	1.3126	0.447	0.0880	0.00553
	3	0.0217	0.438	9.75 · 10^−15^	0.527	0.580	2.10	0.332	0.00682
	4	1100	0.349	8590	0.2537	0.765	1.72	0.141	0.00780
	5	0.346	0.160	0.537	2.81 · 10^−16^	1.31	0.447	0.0880	0.00553
	6	203	0.349	1580	0.254	0.764	1.72	0.141	0.00780
	7	0.0217	0.438	1.51 · 10^−16^	0.527	0.580	2.10	0.332	0.00682
	8	0.0217	0.438	4.65 · 10^−15^	0.527	0.580	2.10	0.332	0.00682
	9	284	0.349	2210	0.254	0.765	1.73	0.141	0.00780
	10	0.0217	0.438	2.75 · 10^−15^	0.527	0.580	2.10	0.332	0.00682
Subject D	1	0.346	0.154	0.432	2.73 · 10^7^	20.3	80.2	0.0550	0.0136
	2	0.346	1.50	0.432	1.45 · 10^9^	15.3	725	68.7	0.0136
	3	0.346	0.159	0.432	4.85 · 10^16^	9.08	37.3	94600	0.0137
	4	0.346	0.173	0.432	1.99 · 10^7^	11.5	52.4	1180	0.0137
	5	0.346	0.102	0.432	2.12 · 10^17^	22.2	50.7	0.00	0.0136
	6	0.344	1.95	0.433	1.16 · 10^7^	3.90	240	208	0.0136
	7	0.346	0.0999	0.432	1.22 · 10^6^	15.4	34.1	3.07	0.0137
	8	0.346	0.951	0.432	2.16 · 10^11^	12.8	379	1710	0.0136
	9	0.134	0.242	0.429	0.435	5.49	37.1	0.00	0.0137
	10	0.347	0.142	0.432	4.14 · 10^8^	163	581	0.00	0.0136
Subject E	1	0.412	0.117	0.361	0.273	0.995	1.02	0.326	0.00550
	2	1.40 · 10^−6^	0.445	142	0.452	0.148	0.693	0.00603	0.00379
	3	2.20 · 10^−13^	0.445	8.71	0.452	0.148	0.692	0.00601	0.00379
	4	0.454	0.0795	0.362	7.12 · 10^−8^	4.51	5.30	1.12	0.00550
	5	0.454	0.0795	0.362	2.97 · 10^−12^	5.40	6.76	1.14	0.00550
	6	0.454	0.0795	0.362	0.0000227	3.41	3.52	1.04	0.00550
	7	0.00	0.454	6.91 · 10^7^	0.419	0.175	0.948	0.0586	0.00402
	8	0.454	0.0795	0.362	0.00	4.11	4.66	1.09	0.00550
	9	0.454	0.0795	0.362	0.0000416	3.21	3.20	1.02	0.00550
	10	0.454	0.0795	0.362	5.41 · 10^−7^	51.2	84.0	1.30	0.00550
Subject F	1	0.456	4.22	0.372	19.3	0.956	1.45 · 10^7^	5.37 · 10^6^	0.00686
	2	1.23 · 10^10^	0.532	4.29 · 10^10^	0.360	1690	15300	0.214	0.00286
	3	0.456	4.21	0.372	14.6	6.23	1.21 · 10^8^	5.22 · 10^6^	0.00686
	4	0.456	4.21	0.372	17.1	1.47	1.70 · 10^7^	3.64 · 10^6^	0.00686
	5	0.456	4.21	0.372	15.4	2.87	1.40 · 10^8^	1.39 · 10^7^	0.00686
	6	0.456	4.21	0.372	17.6	1.28	1.54 · 10^7^	3.88 · 10^6^	0.00686
	7	0.456	4.16	0.372	48.0	0.364	185	407	0.00687
	8	0.456	4.22	0.372	16.1	2.02	1.42 · 10^7^	1.42 · 10^7^	0.00686
	9	4.97 · 10^8^	0.531	1.73 · 10^9^	0.360	33600	304000	0.214	0.00286
	10	0.456	4.21	0.372	15.7	2.51	1.44 · 10^8^	1.65 · 10^7^	0.00686

In some cases the model parameters are estimated to be zero, or very close to zero, for example *k*_34_ for subject B, *k*_12_ and *k*_14_ for subject C, *k*_34_ for subject D, and *k*_21_ and *k*_14_ for subject E. For each of these subjects the data can be well represented by a reduced model in which either IgG-FcRn binding is irreversible (*k*_34_ = 0), there is no transfer from the peripheral compartment to plasma (*k*_12_ = 0) or vice versa (*k*_21_ = 0), or bound IgG molecules are not recycled into plasma (*k*_14_ = 0). This result suggests that the model complexity is not supported by the available data.

**Figure 5:**
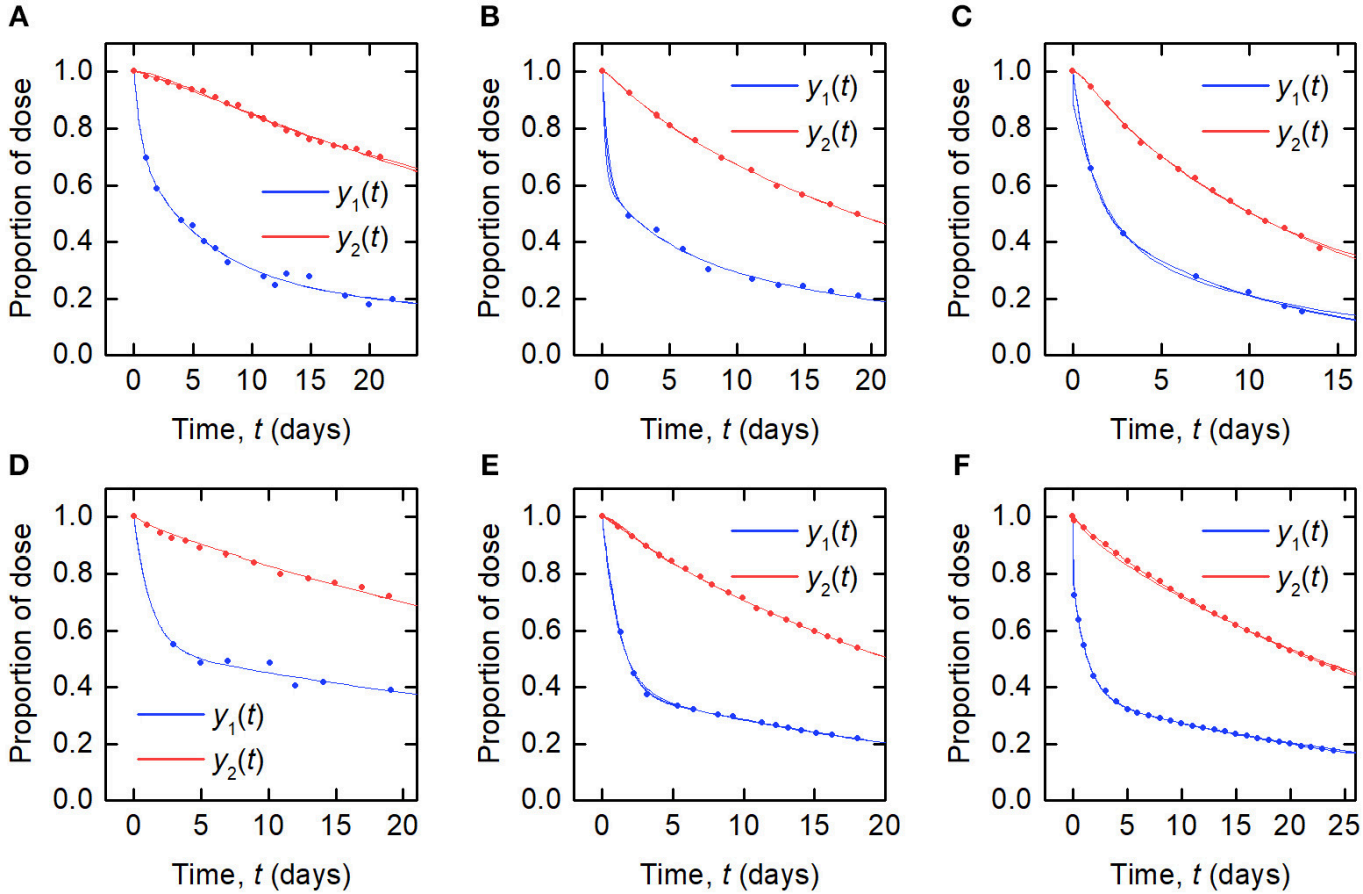
Timecourse data [*y*_1_(*t*) blue circles; *y*_2_(*t*) red circles] and model fits [*y*_1_(*t*) blue line; *y*_2_(*t*) red line] for **(A–F)** subjects A–F.

The data and the model outputs using the parameter estimates in Table 3 are plotted in Figure 5. In each panel of Figure 5, the model outputs *y*_1_(*t*) and *y*_2_(*t*) are plotted for each of the estimated parameter vectors from 10 runs. The model outputs are very similar for all of the estimated parameter vectors for an individual. For some subjects there are small but noticeable differences between the fits, for example: in the first and last 5 days of *y*_2_(*t*) for subject A; in the first 2 days of *y*_1_(*t*) for subject B; for all of *y*_1_(*t*) and the latter part of *y*_2_(*t*) for subject C; between days 2 and 6 for *y*_1_(*t*) and the initial 2 days of *y*_2_(*t*) for subject E; and the first 10 days and final 5 days of *y*_2_(*t*) for subject F. The similarity between the outputs for the parameter estimates obtained across different runs is shown by the similar values of RMSE within each subject. The model appears to fit the data reasonably well and in some subjects extremely well.

The results of the multiple runs of differential evolution show that in many cases, highly different parameter vectors produce very similar model outputs. The spread of the parameter estimates from multiple runs is conveyed using the coefficient of variation (CV), that is, the standard deviation of the estimates of a parameter from 10 runs, divided by the mean of those estimates. The CV is tabulated in Table 4. For some parameters and subjects, the estimates for the parameters have a small CV, for example the first four parameters for subject A and parameter *k*_12_ for subject D. In other instances however the CV is much larger, reflecting the highly different estimates obtained for these parameters. The similarly high quality fits produced by diverse parameter vectors implies that, whilst the parameters are structurally identifiable, they are not all *practically* identifiable for the quality of data that are available.

**Table 4:** Coefficients of variation of parameter estimates obtained from 10 runs of differential evolution, for each of subjects A–F.

**Parameter**	**Coefficient of variation**					
	**A**	**B**	**C**	**D**	**E**	**F**
*k*_21_	0.00404	0.634	2.18	0.206	0.691	3.03
*k*_31_	0.0405	0.905	0.312	1.24	0.907	0.446
*k*_12_	0.0458	0.642	2.18	0.00277	3.16	3.03
*k*_14_	0.0417	1.38	0.643	2.58	1.33	0.792
*k*_03_	0.280	0.461	0.373	1.71	2.12	3.00
*k*_43_	0.360	1.85	0.399	1.15	2.32	1.14
*k*_34_	0.305	3.16	0.502	3.05	0.758	1.01

### 3.2. Parameter Identification Using Fractional Catabolic Rate Data

Authors who have studied a two-compartment model of IgG metabolism have previously estimated parameters from FCR vs. plasma IgG concentration data (16, 21). In this section we investigate whether it is possible to estimate parameters of the four-compartment model from these data, which are described in section 2.2. In section 2.6 two expressions for the FCR were introduced: the FCR of the tracer (Equation 11) and the FCR of the endogenous IgG in steady state (Equation 14). In practice FCR_T_ is measured; however it is difficult to obtain a closed form expression for FCR_T_. In contrast, we can easily obtain an expression for FCR_E_ in terms of the model parameters and the quantity of endogenous IgG in plasma, *x*_1,E_, as given by Equation (14). In this section model parameters are estimated by fitting the expression for FCR_E_ vs. *x*_1,E_ Equation (14) to the FCR_T_ vs. *x*_1,E_ data. It is assumed that FCR_E_ is a good approximation to FCR_T_ and the parameter estimates are validated in section 3.2.3 using synthetic data.

#### 3.2.1. Structural Identifiability Analysis

The relationship between FCR_E_ and *x*_1,E_ is given by Equation (14). Given that the parameters *k*_on_ and *v*_3_ only appear in the model (Equations 3) as the ratio *k*_on_/*v*_3_, we re-write Equation (14), defining ϕ_1_ = *k*_on_/*v*_3_, giving

(21) $$\begin{array}{l} {\text{FCR}}_{\text{E}}=\frac{1}{2\phi_{1}x_{1,\text{E}}}(k_{31}\phi_{1}x_{1,\text{E}}-k_{14}\phi_{1}R_{\text{tot}}-k_{03}k_{14}-k_{03}k_{\text{off}}\\ +\sqrt{4k_{03}k_{31}\left(k_{14}+k_{\text{off}}\right)\phi_{1}x_{1,\text{E}}+{\left(-k_{31}\phi_{1}x_{1,\text{E}}+k_{14}\phi_{1}R_{\text{tot}}+k_{03}\left(k_{14}+k_{\text{off}}\right)\right)}^{2}}). \end{array}$$

We wish to know whether the parameter vector $\phi ={\left(\phi_{1},k_{31},k_{14},R_{\text{tot}},k_{03},k_{\text{off}}\right)}^{\text{T}}$ is structurally identifiable with respect to the relationship in Equation (21). The structural identifiability problem amounts to determining whether there exists an alternative parameter vector $\bar{\phi }={\left({\bar{\phi }}_{1},{\bar{k}}_{31},{\bar{k}}_{14},{\bar{R}}_{\text{tot}},{\bar{k}}_{03},{\bar{k}}_{\text{off}}\right)}^{\text{T}}$ such that ${\text{FCR}}_{\text{E}}\left(x_{1,\text{E}},\phi \right)=\;{\text{FCR}}_{\text{E}}\left(x_{1,\text{E}},\bar{\phi }\right)$.

From Equations (13) and (2),

(22) $$\begin{array}{l} {\text{FCR}}_{\text{E}}=\frac{I_{0}}{{\hat{x}}_{1}}. \end{array}$$

*I*_0_ is given in terms of ${\hat{x}}_{1}$ by the solution of the following quadratic equation, obtained by rearranging the first equation of Equations (2) and setting ϕ_1_ = *k*_on_/*v*_3_:

(23) $$\begin{array}{l} -\phi_{1}I_{0}^{2}+\left(-k_{03}\left(k_{14}+k_{\text{off}}\right)+\phi_{1}\left(k_{31}{\hat{x}}_{1}-k_{14}R_{\text{tot}}\right)\right)I_{0}\\ \;+k_{03}k_{31}\left(k_{14}+k_{\text{off}}\right){\hat{x}}_{1}=0. \end{array}$$

Substituting ${\text{FCR}}_{\text{E}}{\hat{x}}_{1}$ in place of *I*_0_ and setting ${\hat{x}}_{1}=x_{1,\text{E}}$ gives the following quadratic equation in FCR_E_:

(24) $$\begin{array}{l} -\phi_{1}x_{1,\text{E}}^{2}{\text{FCR}}_{\text{E}}^{2}+\left(-k_{03}\left(k_{14}+k_{\text{off}}\right)+\phi_{1}\left(k_{31}x_{1,\text{E}}-k_{14}R_{\text{tot}}\right)\right)\\ x_{1,\text{E}}{\text{FCR}}_{\text{E}}+k_{03}k_{31}\left(k_{14}+k_{\text{off}}\right)x_{1,\text{E}}=0. \end{array}$$

Dividing Equation (24) throughout by the coefficient of ${\text{FCR}}_{\text{E}}^{2}$ gives

(25) $$\begin{array}{l} {\text{FCR}}_{\text{E}}^{2}+\left(\frac{k_{03}\left(k_{14}+k_{\text{off}}\right)-k_{31}\phi_{1}x_{1,\text{E}}+k_{14}\phi_{1}R_{\text{tot}}}{\phi_{1}x_{1,\text{E}}}\right){\text{FCR}}_{\text{E}}\\ -\frac{k_{03}k_{31}\left(k_{14}+k_{\text{off}}\right)}{\phi_{1}x_{1,\text{E}}}=0. \end{array}$$

The expression for FCR_E_ given by Equation (21) is one of the two solutions of Equation (25). We therefore wish to know whether there exists an alternative parameter vector $\bar{\phi }$ such that,

(26) $$\begin{array}{l} \begin{array}{cc} {\text{FCR}}_{\text{E}}^{2}+ & \left(\frac{k_{03}\left(k_{14}+k_{\text{off}}\right)-k_{31}\phi_{1}x_{1,\text{E}}+k_{14}\phi_{1}R_{\text{tot}}}{\phi_{1}x_{1,\text{E}}}\right){\text{FCR}}_{\text{E}}\\ -\frac{k_{03}k_{31}\left(k_{14}+k_{\text{off}}\right)}{\phi_{1}x_{1,\text{E}}}\\ ={\text{FCR}}_{\text{E}}^{2}+\left(\frac{{\bar{k}}_{03}\left({\bar{k}}_{14}+{\bar{k}}_{\text{off}}\right)-{\bar{k}}_{31}{\bar{\phi }}_{1}x_{1,\text{E}}+{\bar{k}}_{14}{\bar{\phi }}_{1}{\bar{R}}_{\text{tot}}}{{\bar{\phi }}_{1}x_{1,\text{E}}}\right)\\ {\text{FCR}}_{\text{E}}-\frac{{\bar{k}}_{03}{\bar{k}}_{31}\left({\bar{k}}_{14}+{\bar{k}}_{\text{off}}\right)}{{\bar{\phi }}_{1}x_{1,\text{E}}}. \end{array} \end{array}$$

From the uniqueness of interpolating polynomials (31, p. 98), the coefficients of the quadratic in Equation (25) are unique, therefore the problem amounts to solving the simultaneous equations:

(27) $$\begin{array}{l} \begin{array}{c} \frac{k_{03}\left(k_{14}+k_{\text{off}}\right)-k_{31}\phi_{1}x_{1,\text{E}}+k_{14}\phi_{1}R_{\text{tot}}}{\phi_{1}x_{1,\text{E}}}\\ =\frac{{\bar{k}}_{03}\left({\bar{k}}_{14}+{\bar{k}}_{\text{off}}\right)-{\bar{k}}_{31}{\bar{\phi }}_{1}x_{1,\text{E}}+{\bar{k}}_{14}{\bar{\phi }}_{1}{\bar{R}}_{\text{tot}}}{{\bar{\phi }}_{1}x_{1,\text{E}}}\\ -\frac{k_{03}k_{31}\left(k_{14}+k_{\text{off}}\right)}{\phi_{1}x_{1,\text{E}}}=-\frac{{\bar{k}}_{03}{\bar{k}}_{31}\left({\bar{k}}_{14}+{\bar{k}}_{\text{off}}\right)}{{\bar{\phi }}_{1}x_{1,\text{E}}}. \end{array} \end{array}$$

The solution was found using the SolveAlways function in Mathematica. The only solution to Equations (27), for all values of *x*_1,E_, is given by

(28) $$\begin{array}{l} \begin{array}{c} {\bar{k}}_{31}=k_{31}\\ {\bar{k}}_{14}{\bar{R}}_{\text{tot}}=k_{14}R_{\text{tot}}\\ \frac{{\bar{k}}_{03}\left({\bar{k}}_{14}+{\bar{k}}_{\text{off}}\right)}{{\bar{\phi }}_{1}}=\frac{k_{03}\left(k_{14}+k_{\text{off}}\right)}{\phi_{1}}. \end{array} \end{array}$$

Therefore, only *k*_31_ and the expressions *k*_14_*R*_tot_ and *k*_03_(*k*_14_+*k*_off_)/ϕ_1_, containing original parameter combinations, are structurally identifiable with respect to the relationship between FCR_E_ and *x*_1,E_.

#### 3.2.2. Parameter Estimation

Having analyzed the structural identifiability of the expression for FCR_E_ vs. *x*_1,E_, it becomes clear that we can rewrite the expression in Equation (14) by combining parameters into new structurally identifiable parameters, as follows:

(29) $$\begin{array}{l} {\text{FCR}}_{\text{E}}(x_{1,\text{E}},\psi )=\frac{1}{2x_{1,\text{E}}}(k_{31}x_{1,\text{E}}-\psi_{1}-\psi_{2}\\ \;+\sqrt{k_{31}^{2}x_{1,\text{E}}^{2}+2k_{31}x_{1,\text{E}}\left(\psi_{1}-\psi_{2}\right)+{\left(\psi_{1}+\psi_{2}\right)}^{2}}), \end{array}$$

where

(30) $$\begin{array}{l} \begin{array}{c} \psi_{1}=\frac{k_{03}v_{3}\left(k_{14}+k_{\text{off}}\right)}{k_{\text{on}}}\\ \psi_{2}=k_{14}R_{\text{tot}} \end{array} \end{array}$$

are uniquely identifiable parameters. ψ_1_ and ψ_2_ have units of μmol day^−1^. The parameter vector to be estimated is now **ψ** = (*k*_31_, ψ_1_, ψ_2_).

It is assumed that Equation (29) is a close approximation to the relationship between the measured FCR_T_ and *x*_1,E_. Waldmann and Strober (21) provide FCR_T_ vs. plasma IgG concentration data. The plasma concentrations of endogenous IgG were multiplied by the average plasma volume *v*_1_, from Table 1, in order to obtain the quantity of endogenous IgG in plasma, *x*_1,E_. The data for FCR_T_ vs. *x*_1,E_ were then fitted using the interior point algorithm implemented within the NonlinearModelFit function in Mathematica. The starting value for the minimization was set to 1 for each parameter. The parameter estimates were constrained to be positive.

**Figure 6:**
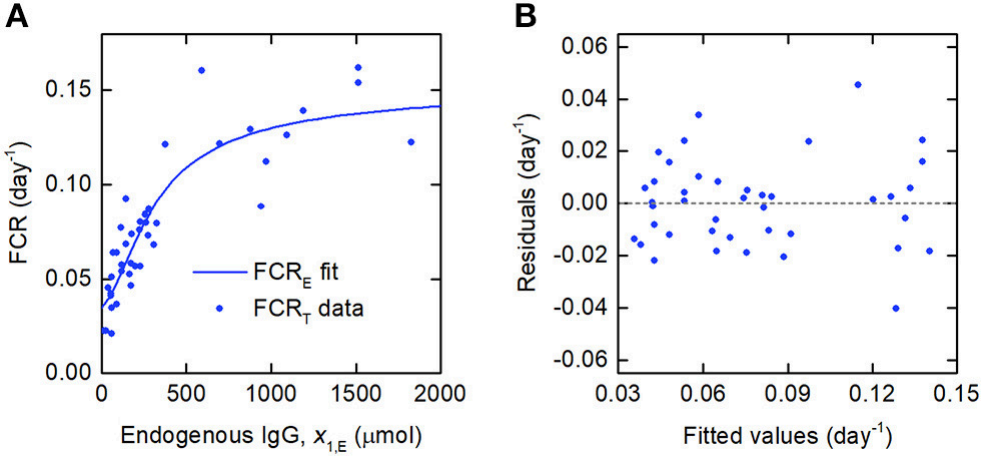
**(A)** Expression for FCR_E_ vs. *x*_1,E_, given by Equation (29), fitted to FCR_T_ vs. *x*_1,E_ data from Waldmann and Strober (21). **(B)** Residuals vs. fitted values.

Since the data were obtained from 41 individuals, the estimated parameter values are assumed to represent the average parameter values within the population. The parameter estimates and their standard errors are provided in Table 5. The fitted expression given by Equation (29) is plotted alongside the data in Figure 6A. The residuals vs. the fitted values are plotted in Figure 6B. On inspection, the model appears to fit the data well. The residuals appear reasonably homoscedastic and there is no obvious autocorrelation.

**Table 5:** Parameter estimates from fitting FCR_E_ expression to FCR_T_ vs. *x*_1,E_ data.

**Parameter**	**Units**	**Estimate**	**Standard error**	**95% confidence interval**
ψ_1_	μmol day^−1^	7.47	2.74	(1.93, 13.0)
ψ_2_	μmol day^−1^	25.7	6.656	(12.3, 39.2)
*k*_31_	day^−1^	0.154	0.00969	(0.135, 0.174)

#### 3.2.3. Validation of Parameter Estimates

There are several issues that may cause the estimates of *k*_31_, ψ_1_, and ψ_2_ to be inaccurate. Firstly, the data were obtained from a sample of 41 individuals, each with their own unique parameter vector; this variability is not accounted for by the estimation procedure. Secondly, the parameters were estimated by fitting the expression for FCR_E_ vs. *x*_1,E_; however the data are for the FCR_T_, which is not equivalent to the FCR_E_. In addition, the FCR_T_ is in practice calculated from measurements of radioactivity in plasma and urine; the form of the measurement errors is therefore not clear.

Due to the aforementioned issues, the validity of the parameter estimates obtained in section 3.2.2 was investigated by estimating the parameters from synthetic data. It is assumed that the parameter values in Table 5 are true population parameter values. Data for FCR_T_ vs. *x*_1,E_ were simulated according to the experimental methodology, described by Waldmann and Strober (21). The data were simulated for 100 sets of 41 subjects. The parameter values were then estimated from the synthetic data, generating 100 estimates for **ψ** = (*k*_31_, ψ_1_, ψ_2_).

In order to simulate the FCR_T_ data, parameter values are required for all model parameters (see Equations 1), not just *k*_31_, ψ_1_, and ψ_2_. Population parameter values are therefore required for all model parameters in order to randomly generate unique parameter vectors for individual subjects. The population parameter values for *k*_21_, *k*_12_, *k*_14_, *k*_03_, and *k*_off_ were fixed to the values from the literature in Table 1. The population value of *k*_31_ was fixed to the estimated value in Table 5. The population values of *R*_tot_ and *k*_on_/*v*_3_ were calculated by substituting the previously fixed parameter values into Equations (30) and solving. In this way, a population parameter vector was found, for which *k*_31_, ψ_1_, and ψ_2_ are equal to their estimated values. Unique parameter values for 41 individuals were randomly generated from a lognormal distribution, with the median given by the population parameter values. The variance was tuned by trial and error in order to replicate the size of the errors seen in the real data. This process was repeated to produce 100 sets of 41 individual parameter vectors and thus 100 sets of FCR_T_ vs. *x*_1,E_ data. Full details of how the synthetic data were generated are provided in the Mathematica code in the Supplementary Material.

**Figure 7:**
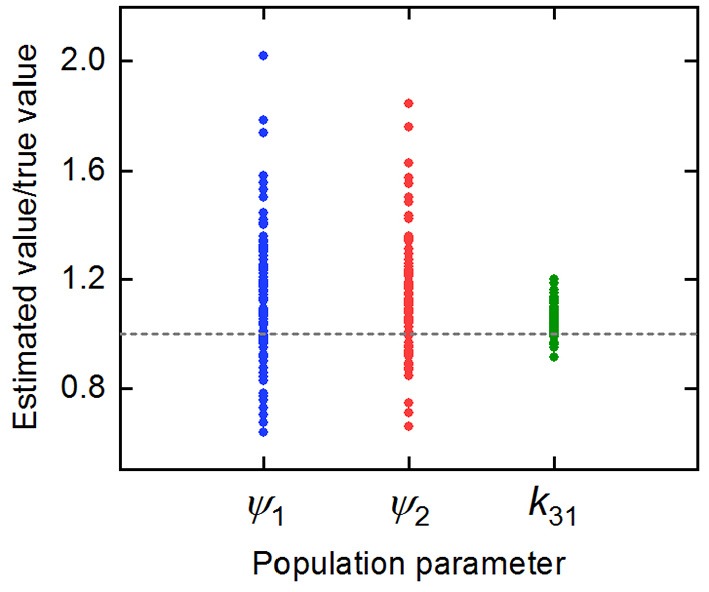
Parameter estimates for *k*_31_, ψ_1_, and ψ_2_ divided by the true parameter value.

The parameter estimates as a proportion of the true parameter values are plotted in Figure 7, showing the spread of the parameter estimates. It is clear from this plot that the parameter *k*_31_ is estimated with higher precision than ψ_1_ and ψ_2_. The sample mean (μ), sample standard deviation (s.d.), bias (*b*), and variability (*v*) of the parameter estimates are given in Table 6. The bias is given by

(31) $$\begin{array}{l} b=\mu -p, \end{array}$$

where *p* is the true value of the parameter. The variability is given by

(32) $$\begin{array}{l} v=\frac{\sqrt{{\text{s.d.}}^{2}+b^{2}}}{p}. \end{array}$$

Variability as defined by Equation (32) has been used by Chen et al. (32) to evaluate the performance of estimation methods when the assumptions relied upon by the methods, in particular relating to noise, are violated. A larger value of *v* represents a worse performance of an estimation method. The results suggest that *k*_31_ has been estimated with a good level of accuracy (*v* = 0.0735), but that the parameters ψ_1_ and ψ_2_ were estimated with a higher level of variability. Based on this result, a future study may look at improving experimental design, for example by increasing the number of subjects, in order to improve upon the variability of the estimates of ψ_1_ and ψ_2_.

**Table 6:** Mean, standard deviation, bias, and variability of the estimates of *k*_31_, ψ_1_, and ψ_2_.

	**Parameter**		
	***k*_31_**	**ψ_1_**	**ψ_2_**
Mean	0.162	8.55	29.0
Standard deviation	0.00758	1.75	5.24
Bias	0.00841	1.08	3.31
Variability	0.0735	0.275	0.241

### 3.3. Simulation of IgG Responses in Multiple Myeloma

It has been shown that parameter estimates obtained using timecourse data are not robust; however, the parameters *k*_31_, ψ_1_, and ψ_2_ may be obtained with reasonably low variability using FCR data. The results from fitting the timecourse data suggest that the model (Equations 1) may be overparameterized with respect to the available data; we therefore ask whether the plasma IgG response can be sufficiently determined using only the parameters *k*_21_, *k*_12_, *k*_31_, ψ_1_, and ψ_2_.

**Figure 8:**
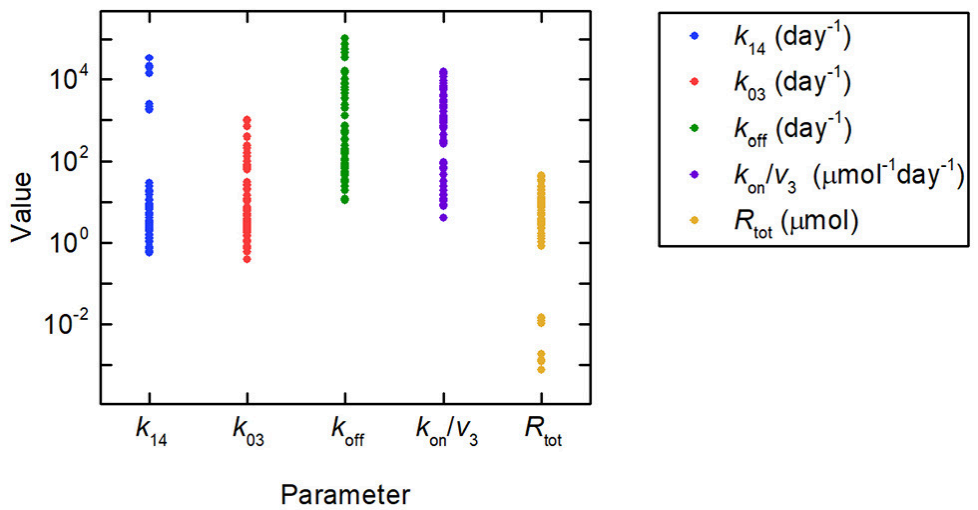
Parameter values used to simulate IgG responses in multiple myeloma, plotted on a logarithmic scale. The method used to generate the parameter values is described in section 3.3. The model predictions generated using these parameter values are shown in Figure 9A. Despite the large amount of variation in the parameter values, the model predictions for plasma IgG are extremely similar.

Firstly we investigate the plasma IgG response given by the full system model (Equations 1), when the parameters *k*_31_, ψ_1_, and ψ_2_ are equal to the values estimated in section 3.2.2. Random values were generated for certain model parameters and the remaining parameter values calculated so that *k*_31_, ψ_1_, and ψ_2_ are equal to their estimated values. Three parameters (not including both *R*_tot_ and *k*_14_) out of *k*_03_, *R*_tot_, *k*_off_, *k*_14_, and *k*_on_/*v*_3_ were fixed to randomly generated values and substituted into Equations (30), yielding a linear system of two equations in two unknowns. Equations (30) were then solved for the remaining two parameters. There are seven sets of three parameters from *k*_03_, *R*_tot_, *k*_off_, *k*_14_, and *k*_on_/*v*_3_, which can be fixed to give the remaining two parameters. Parameters were generated 10 times, as described, for each of these seven sets, giving 70 parameter vectors in total. The randomly generated parameter values were obtained by assuming a lognormal distribution, in order to ensure positivity, with median set to the parameter value from the literature, given in Table 1, and variance 1. The values generated in this way for the parameters *k*_03_, *R*_tot_, *k*_off_, *k*_14_, and *k*_on_/*v*_3_ are depicted in Figure 8, showing the extremely wide range of parameter values used. The parameter *k*_31_ was set to the estimated value given in Table 5. The values for *k*_21_ and *k*_12_ were set to the values given in Table 1.

In order to simulate the model under realistic clinical conditions, a model for the IgG synthesis rate in multiple myeloma was used, which has been found to predict responses consistent with real patient data (33). The IgG synthesis rate is described by

(33) $$\begin{array}{l} I\left(t\right)=\left(I_{0}-I_{\infty }\right)\exp\left(-k_{\text{kill}}t\right)+I_{\infty }. \end{array}$$

The following parameter values were used to produce the simulation: *I*_0_ = 76 μmol day^−1^, *I*_∞_ = 26.5 μmol day^−1^, and *k*_kill_ = 0.055 day^−1^ (33).

**Figure 9:**
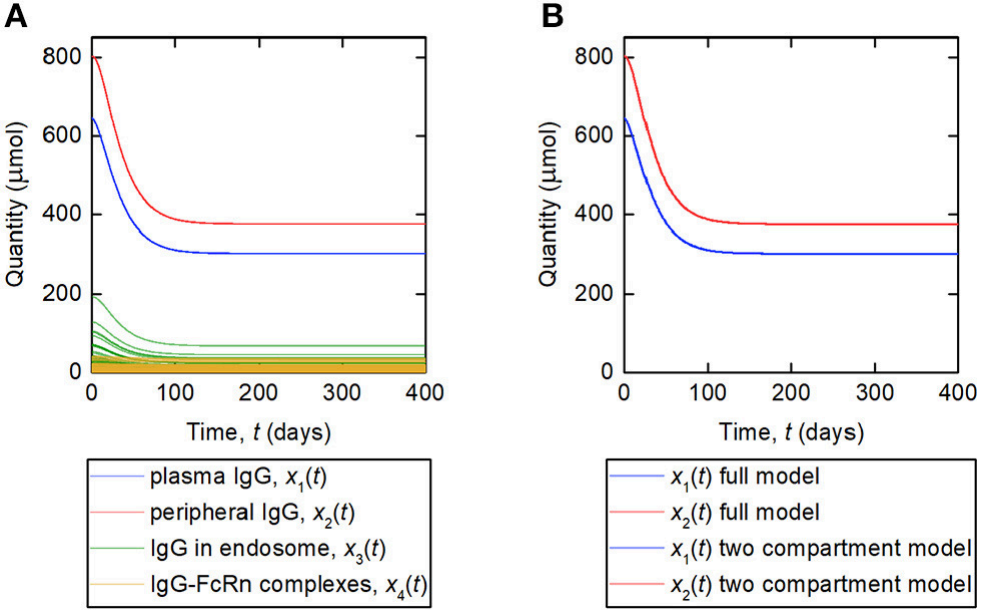
**(A)** Simulation of responses of plasma IgG [(*x*_1_(*t*)], peripheral IgG [*x*_2_(*t*)], IgG in endosomes [*x*_3_(*t*)], and IgG bound to FcRn in endosomes [*x*_4_(*t*)]. The scenario shown represents a decreasing tumor burden during therapy. Each variable is simulated for 70 unique parameter vectors. **(B)** Simulation of responses of plasma IgG [*x*_1_(*t*)], peripheral IgG [*x*_2_(*t*)], compared for the four-compartment model and the proposed two compartment model. The responses are indistinguishable by inspection for the two models.

A simulation of the responses in all four model compartments is shown in Figure 9A. Each variable is simulated for 70 unique parameter vectors. The predicted trajectories for plasma IgG and peripheral IgG, respectively, are extremely similar for all 70 parameter vectors; however there is some variation in the responses of IgG in intracellular endosomes, particularly the IgG that is not bound to FcRn receptors. The simulation suggests that, under the investigated conditions, the response in the plasma compartment is relatively insensitive to changes in the individual parameters *k*_03_, *R*_tot_, *k*_off_, *k*_14_, and *k*_on_/*v*_3_, provided that the parameters *k*_31_, ψ_1_, and ψ_2_ are fixed. The maximal difference between any two trajectories for *x*_1_(*t*) at any simulated time point is 0.2%.

The lack of variation within the predicted responses for plasma and peripheral IgG, when parameters *k*_21_, *k*_12_, *k*_31_, ψ_1_, and ψ_2_ are fixed, suggests that it may be possible to simulate these two variables using a reduced order model based upon the newly derived expression for the FCR (Equation 29). The equations for this model are given by

(34) $$\begin{array}{l} \begin{array}{c} {ẋ}_{1}\left(t\right)=-\left(k_{21}+f\left(x_{1}\left(t\right)\right)\right)x_{1}\left(t\right)+k_{12}x_{2}\left(t\right)+k_{14}x_{4}\left(t\right)+I\left(t\right)\\ {ẋ}_{2}\left(t\right)=k_{21}x_{1}\left(t\right)-k_{12}x_{2}\left(t\right), \end{array} \end{array}$$

where

(35) $$\begin{array}{l} f\left(x_{1}\left(t\right)\right)=\frac{1}{2x_{1}(t)}(k_{31}x_{1}(t)-\psi_{1}-\psi_{2}\\ \;+\sqrt{k_{31}^{2}x_{1}{\left(t\right)}^{2}+2k_{31}x_{1}(t)\left(\psi_{1}-\psi_{2}\right)+{\left(\psi_{1}+\psi_{2}\right)}^{2}}). \end{array}$$

The assumption behind this model is that the fractional rate of IgG catabolism is equal to its fractional rate of catabolism at steady state. A simulation of this model, alongside the original four-compartment model, is shown in Figure 9B. The model is simulated with values of *k*_21_ and *k*_12_ from Table 1 and all other parameter values from Table 5. The responses for *x*_1_(*t*) and *x*_2_(*t*) are very similar for the two models and appear overlayed in Figure 9B. The maximal difference between *x*_1_(*t*) predicted by the two-compartment model and *x*_1_(*t*) predicted by the four-compartment model, for any of the 70 parameter vectors used and at any simulated time point, is 0.2%. The responses are indistinguishable by inspection for the two models.

The proposed two-compartment model is based upon the assumption that the fractional rate of IgG catabolism is equal to its fractional rate of catabolism in steady state. When the system is in steady state, this assumption is of course true. However, faster dynamics, caused by a rapid change in the IgG synthesis rate, will cause this assumption to progressively weaken. Further study of the proposed model is required to analyse its relationship with the original four-compartment model and to determine under what conditions the proposed model predictions are within an acceptable region of the four-compartment model predictions.

## 4. Discussion

The motivation behind the research presented in this paper was to investigate a model suitable for predicting IgG responses in patients with IgG multiple myeloma. When producing predictive simulations of a biomedical system, it is important to know the level of confidence in the model parameter values.

There are numerous published models of FcRn-mediated recycling of IgG in the literature, some of which are cited in the Introduction. Most of these models were developed for IgG-based therapeutic monoclonal antibodies and may not be suitable for characterizing endogenous IgG. Those models characterizing endogenous IgG, for example the models of Li et al. (34) and Chen and Balthasar (10), rely upon a mixture of animal and human data for sourcing parameter values.

For example, parameter values provided by Li et al. (34) for endogenous IgG were taken from the literature, apart from the catabolic clearance (corresponding to *k*_03_ of the present study), the vascular reflection coefficient (not included in our model), and the recycling rate constant (corresponding to *k*_14_ of the present study). These parameter values were obtained by manually varying the parameters within the model until the results showed a mean half-life of 21 days, a mean IgG synthesis rate of 34 mg kg^−1^ day^−1^ and a realistic fold reduction in IgG concentration when FcRn is not present. The values used for the half-life and synthesis rate are those obtained from normal human data by Waldmann and Strober (21) and Waldmann and Terry (23).

One of the problems with the approach taken in previous papers is that, whilst parameter values have been found that provide a half-life of 21 days for an IgG synthesis rate of 34 mg kg^−1^ day^−1^, it is not clear what would happen to the half-life when the IgG synthesis rate increases or decreases, under the obtained parameter values. This approach is therefore akin to fitting a model to a curve having only one data point. The nonlinear relationship between synthesis or concentration of IgG and its half-life, which is fundamental to the FcRn-IgG recycling system, may therefore not be captured accurately using this approach.

Another issue with this earlier approach is that it requires the parameter values obtained from the literature to be fixed while the remaining values are varied, therefore implicitly assuming complete confidence in the fixed parameter values that were sourced in the literature. One would question what would happen if one or more of these parameter values were inaccurate by, say, 10% or more, what would be the effect on the corresponding values obtained for *k*_14_ and *k*_03_?

Having considered the models available in the literature and their issues in respect of parameter identifiability, we identified the need for a semi-mechanistic model with parameter values obtained using only *in vivo* human data. This approach necessitated a simpler model than those available in the literature and previously discussed. The model studied in this paper is therefore missing some of the mechanisms of the more complex models. However, simplified compartmental models can often be derived from complex physiologically-based models by lumping compartments and processes. Lumped models may be adequate for describing processes of interest, for example responses in a central/plasma compartment. Fronton et al. (14) demonstrate the correspondence between a physiologically-based model and several compartmental model structures for IgG. A similar study could be performed using the models presented in this paper in future work.

In this paper, two observed model outputs were considered: the timecourse of the proportion of a dose of IgG remaining in plasma and in the body of an individual subject; and the FCR vs. the quantity of endogenous IgG in plasma, measured in a cohort of subjects with a range of plasma IgG concentrations. We derived mathematical descriptions of these experimental observations based on the underlying model. Structural identifiability analysis was performed with respect to these observations in order to determine which parameters are structurally uniquely identifiable from the available outputs.

In section 3.1 we estimated parameter values using data for the timecourse of an administered dose of radiolabeled IgG in plasma and in the body. We found that all parameters of the linearized model are structurally globally identifiable. Whilst the model is capable of fitting the data well, the results of 10 runs of differential evolution suggest that the parameter estimates are not robust. Highly different parameter vectors, as illustrated by the relative standard deviations of parameter estimates from 10 runs, produce similarly excellent fits to the data. These results suggest that the available data do not support the complexity of the model. A future study may apply a systematic analysis of model sensitivity and parameter correlations, for example using the profile-likelihood method of Raue et al. (35) or generalized sensitivity functions of Thomaseth and Cobelli (36) [extended to multiple output models by Kappel and Munir (37)]. Another potential study for future work could involve estimating model parameters from synthetic timecourse data, to see whether more frequent sampling or a longer observation period provides more stable parameter estimates. However, as highly different parameter values produce similarly excellent fits to the data, the type of data needed for robust parameter estimation is likely to be of a very high quality. As the data are obtained by taking blood samples, there is a practical limitation on the sampling frequency for an individual subject.

The data used were obtained from tracer experiments that were performed between 1963 and 1990. More recent IgG timecourse data are available; however, these data pertain to therapeutic monoclonal antibodies, which can have different kinetics (38). Timecourse data are also available for patients with IgG multiple myeloma, whose serum IgG concentration is monitored during therapy. However, the production rate of IgG in these patients is determined by the status of the disease. Using these data to estimate model parameters would therefore require simultaneous estimation of IgG production parameters. This would require a more complex structural identifiability analysis and may be considered in future work. For these reasons, more recent data were not used in this study.

The structural identifiability of the relationship between FCR_E_ and the quantity of endogenous IgG in plasma, *x*_1,E_, was analyzed. We found that the parameter *k*_31_ and newly defined parameters ψ_1_ = (*k*_03_*v*_3_(*k*_14_ + *k*_off_))/*k*_on_ and ψ_2_ = *k*_14_*R*_tot_ are structurally globally identifiable. These new parameters were estimated using least squares estimation. Estimation with synthetic data shows that these parameters can be estimated with a reasonable level of variability. The parameters *k*_31_ and ψ_2_ are physiologically meaningful: *k*_31_ is the rate at which plasma IgG is internalized into intracellular endosomes and ψ_2_ is the maximal rate of recycling of IgG from endosomes into plasma. The 95% confidence interval for *k*_31_ (0.135–0.174 day^−1^) is similar to other values reported in the literature [0.13 day^−1^ (17); 0.18 day^−1^ (21); 0.16 day^−1^ (33)]. The 95% confidence interval for ψ_2_ (12.3–39.2 μmol day^−1^) is smaller than previously reported values [68.6 μmol day^−1^ (16); 103 μmol day^−1^ (17)]; however it overlaps with the 95% confidence interval (19.1–60.9 μmol day^−1^) reported by Kendrick et al. (33).

In applications in which the behavior of the variables *x*_3_(*t*) and *x*_4_(*t*), representing unbound and bound IgG in intracellular endosomes, respectively, are of great importance, clearly parameter values are required which determine their behavior, including receptor-ligand binding (*k*_on_/*v*_3_, *k*_off_, and *R*_tot_), recycling of bound IgG into plasma (*k*_14_) and degradation of unbound IgG (*k*_03_). The results presented in this paper suggest that it is not possible to estimate these parameters from the available data that are only based upon measurements in plasma. In section 3.3, it is shown that these parameters can be varied by several orders of magnitude (see Figure 8) whilst having a minimal effect on the plasma IgG response (see Figure 9A). It is possible that the actions of the parameters determining recycling, degradation, association and dissociation can approximately balance each other out with respect to the dynamics in the plasma compartment, even though the responses of IgG in the endosome are affected by changes in these parameter values. For investigations limited to the behavior of IgG in plasma, model reduction using the parameters *k*_31_, ψ_1_, and ψ_2_ could be investigated in future work. A two-compartment model based upon the newly derived expression for the FCR has been proposed in section 3.3. Further analysis of this model is required to determine whether it is suitable for investigating IgG responses under a range of clinical conditions.

In future work the models studied in this paper could be used to simulate plasma IgG responses in clinical applications, such as the bone marrow cancer multiple myeloma, in which malignant plasma cells secrete large quantities of monoclonal Ig (M-protein). It has been suggested that the FcRn-IgG interaction may play a significant role in the detection of M-protein using a recently-developed mass spectrometry-based method (4). It was found that in patients with IgG-producing disease, the test result was more likely to be positive for M-protein after three months than in patients with IgA-producing disease, whereas after 12 months the patients were equally likely to have a positive test result. Mills et al. (4) have suggested that this effect is due to FcRn-mediated recycling extending the half-life of IgG, emphasizing the importance of assessment times of response. FcRn-mediated recycling also plays a role in the pharmacokinetics of the novel monoclonal IgG agent for multiple myeloma, daratumumab. Yan et al. (5) found that the isotype of the patient's M-protein has an effect on drug exposure, with IgG patients having significantly lower daratumumab concentrations than patients with other M-protein types. Yan et al. (5) proposed that competition between the IgG M-protein and IgG-based daratumumab for FcRn receptors is the reason for this phenomenon. These recent studies show the importance of FcRn-mediated recycling of IgG in multiple myeloma and the need for mathematical modeling and simulation of this system. The model studied in this paper could be used in future work to investigate such problems.

There is a trade-off in modeling between model accuracy, which is more often represented in complex physiologically-based pharmacokinetic models, and accuracy of parameter values, which is more easily achieved with simplified compartmental models. At present, there are very few studies available on parameter estimation for models of IgG-FcRn kinetics using human data due to issues of parameter identifiability. This paper not only provides useful parameter estimates and suggests a novel model structure, but also exposes some of the difficulties in achieving this aim. Researchers pursuing physiologically-based models of IgG in the future may find it useful to compare the rate of IgG internalization into endosomes and the maximal rate of IgG recycling in their model with the values that we have estimated from human data [considering the approach of Li et al. (34) discussed above]. Furthermore, our paper shows the level of analysis (including structural identifiability analysis, estimation from synthetic data, for example) required in order to have confidence in parameter estimates obtained and an understanding of their meaning to the model.

## 5. Conclusion

It is not possible to estimate all of the model parameters robustly; however certain structurally identifiable parameter combinations have been estimated with a good level of variability. Plasma IgG responses, under typical clinical conditions, are insensitive to large changes in many of the model parameters, provided that certain parameters and parameter combinations are fixed. A reduced-order model, based upon the newly derived expression for the FCR, shows potential for simulating plasma IgG responses under clinical conditions.

## Author Contributions

FK performed model analyses. FK, MC, and NE wrote the manuscript. SH initiated the work. MC, NE, OB, and SH supervised the work. SH and OB provided discussion on the clinical application of the work. All authors reviewed and approved the final manuscript.

### Conflict of Interest Statement

OB and SH were employed by company The Binding Site Limited. The remaining authors declare that the research was conducted in the absence of any commercial or financial relationships that could be construed as a potential conflict of interest.
