# Supplementary material for: Parameter Identification for a Model of Neonatal Fc Receptor-Mediated Recycling of Endogenous Immunoglobulin G in Humans
Source: Front Immunol. 2019 Apr 8;10:674. doi: 10.3389/fimmu.2019.00674 (PMC6465738; doi:10.3389/fimmu.2019.00674)
Supplement: Supplementary file 1 [file Data_Sheet_1.pdf]

Supplementary Material: Parameter identification for a model of neonatal Fc receptor-mediated recycling of endogenous immunoglobulin G in humans for clinical application

1 STABILITY ANALYSIS

Linearising the system about the equilibrium point gives

$$\begin{pmatrix}\dot{x}_1(t)\\ \dot{x}_2(t)\\ \dot{x}_3(t)\\ \dot{x}_4(t)\end{pmatrix} = \begin{pmatrix} -k_{21}-k_{31} & k_{12} & 0 & k_{14} \\ k_{21} & -k_{12} & 0 & 0 \\ k_{31} & 0 & a_{33} & k_{\text{off}} + \frac{k_{\text{on}} I_0}{k_{03} v_3} \\ 0 & 0 & a_{43} & -k_{14} - k_{\text{off}} - \frac{k_{\text{on}} I_0}{k_{03} v_3} \end{pmatrix} \begin{pmatrix} x_1(t)\\ x_2(t)\\ x_3(t)\\ x_4(t)\end{pmatrix}, \tag{S1}$$

where

$$\begin{aligned} a_{33} &= -\frac{k_{03}(k_{\text{on}}(I_0 + (k_{14}+k_{\text{off}})\,R_{\text{tot}}) + k_{03} v_3\,(k_{14}+k_{\text{off}})}{k_{\text{on}} I_0 + k_{03} v_3\,(k_{14}+k_{\text{off}})} \\ a_{43} &= \frac{k_{03} k_{\text{on}} R_{\text{tot}}\,(k_{14}+k_{\text{off}})}{k_{\text{on}} I_0 + k_{03} v_3\,(k_{14}+k_{\text{off}})}. \end{aligned} \tag{S2}$$

The coefficients of the characteristic polynomial are given by

$$c_1 = \frac{1}{k_{03} v_3\big(k_{\text{on}} I_0 + k_{03} v_3 (k_{14}+k_{\text{off}})\big)} \Big(I_0^2 k_{\text{on}}^2 + I_0 k_{03}(k_{03}+k_{12}+2k_{14}+k_{21}+k_{31}+2k_{\text{off}}) k_{\text{on}} v_3 \\ + k_{03}^2 (k_{14}+k_{\text{off}}) v_3 \big(k_{\text{on}} R_{\text{tot}} + (k_{03}+k_{12}+k_{14}+k_{21}+k_{31}+k_{\text{off}}) v_3\big)\Big)$$

$$c_2 = \frac{1}{k_{03} v_3\big(k_{\text{on}} I_0 + k_{03} v_3 (k_{14}+k_{\text{off}})\big)} \bigg(I_0^2 (k_{03}+k_{12}+k_{21}+k_{31}) k_{\text{on}}^2 + I_0 k_{03}\big(2(k_{21}+k_{31})(k_{14}+k_{\text{off}}) \\ + k_{12}(2k_{14}+k_{31}+2k_{\text{off}}) + k_{03}(k_{12}+2k_{14}+k_{21}+k_{31}+2k_{\text{off}})\big) k_{\text{on}} v_3 \\ + k_{03}^2 (k_{14}+k_{\text{off}}) v_3 \Big(k_{21} k_{\text{on}} R_{\text{tot}} + k_{31} k_{\text{on}} R_{\text{tot}} + k_{03} k_{21} v_3 + k_{03} k_{31} v_3 + k_{03} k_{\text{off}} v_3 + k_{21} k_{\text{off}} v_3 \\ + k_{31} k_{\text{off}} v_3 + k_{14}\big(k_{\text{on}} R_{\text{tot}} + (k_{03}+k_{21}+k_{31}) v_3\big) + k_{12}\big(k_{\text{on}} R_{\text{tot}} + (k_{03}+k_{14}+k_{31}+k_{\text{off}}) v_3\big)\Big)\bigg)$$

$$c_3 = \frac{1}{k_{03}v_3\big(k_{\text{on}}I_0 + k_{03}v_3(k_{14}+k_{\text{off}})\big)}\Bigg(I_0^2\big(k_{12}k_{31} + k_{03}(k_{12}+k_{21}+k_{31})\big)k_{\text{on}}^2$$
$$+ I_0k_{03}\Big(2k_{12}k_{31}(k_{14}+k_{\text{off}}) + k_{03}\big(2(k_{21}+k_{31})(k_{14}+k_{\text{off}}) + k_{12}(2k_{14}+k_{31}+2k_{\text{off}})\big)\Big)k_{\text{on}}v_3$$
$$+ k_{03}^2(k_{14}+k_{\text{off}})v_3\Bigg(k_{03}(k_{21}+k_{31})k_{\text{off}}v_3 + k_{14}(k_{21}k_{\text{on}}R_{\text{tot}} + k_{03}k_{21}v_3 + k_{03}k_{31}v_3) + k_{12}\Big(k_{03}k_{\text{off}}v_3$$
$$+ k_{14}\big(k_{\text{on}}R_{\text{tot}} + (k_{03}+k_{31})v_3\big) + k_{31}\big(k_{\text{on}}R_{\text{tot}} + (k_{03}+k_{\text{off}})v_3\big)\Big)\Bigg)\Bigg)$$

$$c_4 = \frac{k_{12}k_{31}\left(k_{03}v_3\left(k_{14}+k_{\text{off}}\right) + k_{\text{on}}I_0\right)}{v_3} \tag{S3}$$

According to the Routh-Hurwitz stability criterion, the four-state system is stable provided the coefficients of the characteristic polynomial of the linearised system satisfy the conditions:

$$\begin{aligned} c_1, c_3, c_4 &> 0 \\ c_1c_2c_3 &> c_3^2 + c_1^2c_4. \end{aligned} \tag{S4}$$

All parameters, including the input I_0, can only take positive values. The conditions given by the first inequality in (S4) can easily be checked by noticing that none of the terms in equations (S3) are preceded by a minus sign. The second inequality was tested in Mathematica using the FullSimplify function. All conditions are found to be met, showing that the equilibrium point of the system is stable.

2 DERIVATION OF LINEAR MODEL OF TRACER DYNAMICS

The dynamics of endogenous IgG and FcRn receptors are described by

$$\begin{aligned} \dot{x}_{1,\text{E}}(t) &= -(k_{21}+k_{31})x_{1,\text{E}}(t) + k_{12}x_{2,\text{E}}(t) + k_{14}x_{4,\text{E}}(t) + I_{\text{E}} \\ \dot{x}_{2,\text{E}}(t) &= k_{21}x_{1,\text{E}}(t) - k_{12}x_{2,\text{E}}(t) \\ \dot{x}_{3,\text{E}}(t) &= k_{31}x_{1,\text{E}}(t) - k_{03}x_{3,\text{E}}(t) - \frac{k_{\text{on}}}{v_3}x_{3,\text{E}}(t)x_{5,\text{E}}(t) + k_{\text{off}}x_{4,\text{E}}(t) \\ \dot{x}_{4,\text{E}}(t) &= \frac{k_{\text{on}}}{v_3}x_{3,\text{E}}(t)x_{5,\text{E}}(t) - (k_{14}+k_{\text{off}})x_{4,\text{E}}(t) \\ \dot{x}_{5,\text{E}}(t) &= -\frac{k_{\text{on}}}{v_3}x_{3,\text{E}}(t)x_{5,\text{E}}(t) + (k_{14}+k_{\text{off}})x_{4,\text{E}}(t). \end{aligned} \tag{S5}$$

where $x_{i,\text{E}}(t)$, $i = 1, \ldots, 4$, is the quantity of unlabelled IgG in compartment i and $x_{5,\text{E}}(t)$ is the quantity of unbound FcRn receptors in intracellular endosomes. The total quantity of FcRn, $x_{4,\text{E}}(t) + x_{5,\text{E}}(t)$, is constant and given by R_{tot}; we have included the equation for the rate of change of $x_{5,\text{E}}(t)$ in (S5) for clarity. Prior to administration of the tracer dose, the system is assumed to be in steady state, where $\hat{x}_i$,

$i = 1, \ldots, 4$, is the quantity of IgG in compartment i in steady state, as given by

$$
\begin{aligned}
\hat{x}_1 &= \frac{I_0\left(k_{03}k_{14}v_3 + k_{03}k_{\text{off}}v_3 + k_{\text{on}}I_0 + k_{14}k_{\text{on}}R_{\text{tot}}\right)}{k_{31}\left(k_{03}v_3(k_{14}+k_{\text{off}}) + k_{\text{on}}I_0\right)} \\
\hat{x}_2 &= \frac{k_{21}}{k_{12}}\hat{x}_1 \\
\hat{x}_3 &= \frac{I_0}{k_{03}} \\
\hat{x}_4 &= \frac{k_{\text{on}}I_0R_{\text{tot}}}{k_{03}v_3(k_{14}+k_{\text{off}}) + k_{\text{on}}I_0},
\end{aligned}
\tag{S6}
$$

with I_0 set to I_{E}. The steady state of unbound FcRn is given by $\hat{x}_5 = R_{\text{tot}} - \hat{x}_4$. For convenience we re-write (S5) as follows:

$$
\begin{aligned}
\dot{x}_{1,\text{E}}(t) &= F_1(x_{1,\text{E}}(t), x_{2,\text{E}}(t), x_{3,\text{E}}(t), x_{4,\text{E}}(t), x_{5,\text{E}}(t)) + I_{\text{E}} \\
\dot{x}_{2,\text{E}}(t) &= F_2(x_{1,\text{E}}(t), x_{2,\text{E}}(t), x_{3,\text{E}}(t), x_{4,\text{E}}(t), x_{5,\text{E}}(t)) \\
\dot{x}_{3,\text{E}}(t) &= F_3(x_{1,\text{E}}(t), x_{2,\text{E}}(t), x_{3,\text{E}}(t), x_{4,\text{E}}(t), x_{5,\text{E}}(t)) \\
\dot{x}_{4,\text{E}}(t) &= F_4(x_{1,\text{E}}(t), x_{2,\text{E}}(t), x_{3,\text{E}}(t), x_{4,\text{E}}(t), x_{5,\text{E}}(t)) \\
\dot{x}_{5,\text{E}}(t) &= F_5(x_{1,\text{E}}(t), x_{2,\text{E}}(t), x_{3,\text{E}}(t), x_{4,\text{E}}(t), x_{5,\text{E}}(t)).
\end{aligned}
\tag{S7}
$$

We treat the administration of a small bolus dose of labelled IgG as a perturbation of the steady state at time $t = 0$. We denote the input of tracer by $u(t)$. Now the dynamics of the total quantity of both labelled and unlabelled IgG are described by

$$
\begin{aligned}
\dot{q}_1(t) &= -(k_{21}+k_{31})q_1(t) + k_{12}q_2(t) + k_{14}q_4(t) + I_{\text{E}} + u(t) \\
\dot{q}_2(t) &= k_{21}q_1(t) - k_{12}q_2(t) \\
\dot{q}_3(t) &= k_{31}q_1(t) - k_{03}q_3(t) - \frac{k_{\text{on}}}{v_3}q_3(t)x_{5,\text{E}}(t) + k_{\text{off}}q_4(t) \\
\dot{q}_4(t) &= \frac{k_{\text{on}}}{v_3}q_3(t)x_{5,\text{E}}(t) - (k_{14}+k_{\text{off}})q_4(t) \\
\dot{x}_{5,\text{E}}(t) &= -\frac{k_{\text{on}}}{v_3}q_3(t)x_{5,\text{E}}(t) + (k_{14}+k_{\text{off}})q_4(t),
\end{aligned}
\tag{S8}
$$

where $q_i(t) = x_{i,\text{E}}(t) + x_{i,\text{T}}(t)$, $i = 1, \ldots, 4$ is the total quantity of labelled and unlabelled IgG in compartment i.

Assuming that the tracer dose is sufficiently small, we have $x_{i,\text{T}}(t) = q_i(t) - \hat{x}_i$, $i = 1, \ldots, 4$, where $x_{i,\text{T}}(t)$ is the quantity of tracer, $q_i(t)$ is the total quantity of tracer and endogenous IgG, and $\hat{x}_i$ is the quantity of IgG in steady state, in compartment i, respectively. Since the FcRn receptors are endogenous to the system they are assumed to be in steady state, such that $x_{5,\text{E}}(t) = \hat{x}_5$, where $\hat{x}_5 = R_{\text{tot}} - \hat{x}_4$ is the quantity of unbound FcRn receptors in equilibrium. The rate of change of the total quantity of IgG in compartment i is given by

$$
\dot{q}_i(t) = \frac{\mathrm{d}}{\mathrm{d}t}\left(x_{i,\text{T}}(t) + \hat{x}_i\right) = \dot{x}_{i,\text{T}}(t), \qquad i = 1, \ldots, 4. \tag{S9}
$$

From (S8) and (S9) we have

$$
\begin{aligned}
\dot{x}_{1,\mathrm{T}}(t) &= -(k_{21}+k_{31})(x_{1,\mathrm{T}}(t)+\hat{x}_1) + k_{12}(x_{2,\mathrm{T}}(t)+\hat{x}_2) + k_{14}(x_{4,\mathrm{T}}(t)+\hat{x}_4) + I_{\mathrm{E}} + u(t)\\
\dot{x}_{2,\mathrm{T}}(t) &= k_{21}(x_{1,\mathrm{T}}(t)+\hat{x}_1) - k_{12}(x_{2,\mathrm{T}}(t)+\hat{x}_2)\\
\dot{x}_{3,\mathrm{T}}(t) &= k_{31}(x_{1,\mathrm{T}}(t)+\hat{x}_1) - k_{03}(x_{3,\mathrm{T}}(t)+\hat{x}_3) - \frac{k_{\mathrm{on}}}{v_3}(x_{3,\mathrm{T}}(t)+\hat{x}_3)\hat{x}_5 + k_{\mathrm{off}}(x_{4,\mathrm{T}}(t)+\hat{x}_4)\\
\dot{x}_{4,\mathrm{T}}(t) &= \frac{k_{\mathrm{on}}}{v_3}(x_{3,\mathrm{T}}(t)+\hat{x}_3)\hat{x}_5 - (k_{14}+k_{\mathrm{off}})(x_{4,\mathrm{T}}(t)+\hat{x}_4).
\end{aligned}
\tag{S10}
$$

We can now approximate the right hand sides of (S10) using the Taylor series expansion. We re-write (S10) as

$$
\begin{aligned}
\dot{x}_{1,\mathrm{T}}(t) &= F_1(\hat{x}_1+x_{1,\mathrm{T}}(t), \hat{x}_2+x_{2,\mathrm{T}}(t), \hat{x}_3+x_{3,\mathrm{T}}(t), \hat{x}_4+x_{4,\mathrm{T}}(t), \hat{x}_5) + I_{\mathrm{E}} + u(t)\\
\dot{x}_{2,\mathrm{T}}(t) &= F_2(\hat{x}_1+x_{1,\mathrm{T}}(t), \hat{x}_2+x_{2,\mathrm{T}}(t), \hat{x}_3+x_{3,\mathrm{T}}(t), \hat{x}_4+x_{4,\mathrm{T}}(t), \hat{x}_5)\\
\dot{x}_{3,\mathrm{T}}(t) &= F_3(\hat{x}_1+x_{1,\mathrm{T}}(t), \hat{x}_2+x_{2,\mathrm{T}}(t), \hat{x}_3+x_{3,\mathrm{T}}(t), \hat{x}_4+x_{4,\mathrm{T}}(t), \hat{x}_5)\\
\dot{x}_{4,\mathrm{T}}(t) &= F_4(\hat{x}_1+x_{1,\mathrm{T}}(t), \hat{x}_2+x_{2,\mathrm{T}}(t), \hat{x}_3+x_{3,\mathrm{T}}(t), \hat{x}_4+x_{4,\mathrm{T}}(t), \hat{x}_5).
\end{aligned}
\tag{S11}
$$

The Taylor series expansion of the right hand side of (S11) is given by

$$
\begin{aligned}
&F_i(\hat{x}_1+x_{1,\mathrm{T}}(t), \hat{x}_2+x_{2,\mathrm{T}}(t), \hat{x}_3+x_{3,\mathrm{T}}(t), \hat{x}_4+x_{4,\mathrm{T}}(t), \hat{x}_5)\\
&\qquad = F_i(\hat{x}_1,\hat{x}_2,\hat{x}_3,\hat{x}_4,\hat{x}_5) + x_{1,\mathrm{T}}(t)\frac{\partial F_i}{\partial x_{1,\mathrm{E}}} + x_{2,\mathrm{T}}(t)\frac{\partial F_i}{\partial x_{2,\mathrm{E}}}\\
&\qquad\quad + x_{3,\mathrm{T}}(t)\frac{\partial F_i}{\partial x_{3,\mathrm{E}}} + x_{4,\mathrm{T}}(t)\frac{\partial F_i}{\partial x_{4,\mathrm{E}}} + \text{H.O.T},
\end{aligned}
\tag{S12}
$$

where H.O.T are higher order terms. Expanding (S11) using (S12) gives

$$
\begin{aligned}
\dot{x}_{1,\mathrm{T}}(t) &\approx -(k_{21}+k_{31})x_{1,\mathrm{T}}(t) + k_{12}x_{2,\mathrm{T}}(t) + k_{14}x_{4,\mathrm{T}}(t) + u(t)\\
\dot{x}_{2,\mathrm{T}}(t) &\approx k_{21}x_{1,\mathrm{T}}(t) - k_{12}x_{2,\mathrm{T}}(t)\\
\dot{x}_{3,\mathrm{T}}(t) &\approx k_{31}x_{1,\mathrm{T}}(t) - k_{03}x_{3,\mathrm{T}}(t) - \frac{k_{\mathrm{on}}}{v_3}\hat{x}_5 x_{3,\mathrm{T}}(t) + k_{\mathrm{off}}x_{4,\mathrm{T}}(t)\\
\dot{x}_{4,\mathrm{T}}(t) &\approx \frac{k_{\mathrm{on}}}{v_3}\hat{x}_5 x_{3,\mathrm{T}}(t) - (k_{14}+k_{\mathrm{off}})x_{4,\mathrm{T}}(t).
\end{aligned}
\tag{S13}
$$

We assume that the tracer dose is small enough that we can neglect higher order terms in the Taylor series expansion. We also recall that $\hat{x}_5 = R_{\mathrm{tot}} - \hat{x}_4$. Finally we obtain the linear model for the tracer dynamics given by

$$
\begin{aligned}
\dot{x}_{1,\mathrm{T}}(t) &= -(k_{21}+k_{31})x_{1,\mathrm{T}}(t) + k_{12}x_{2,\mathrm{T}}(t) + k_{14}x_{4,\mathrm{T}}(t) + u(t)\\
\dot{x}_{2,\mathrm{T}}(t) &= k_{21}x_{1,\mathrm{T}}(t) - k_{12}x_{2,\mathrm{T}}(t)\\
\dot{x}_{3,\mathrm{T}}(t) &= k_{31}x_{1,\mathrm{T}}(t) - k_{03}x_{3,\mathrm{T}}(t) - k_{43}x_{3,\mathrm{T}}(t) + k_{34}x_{4,\mathrm{T}}(t)\\
\dot{x}_{4,\mathrm{T}}(t) &= k_{43}x_{3,\mathrm{T}}(t) - (k_{14}+k_{34})x_{4,\mathrm{T}}(t),
\end{aligned}
\tag{S14}
$$

where k_{34} and k_{43} are given by

$$\begin{aligned} k_{34} &= k_{\text{off}} \\ k_{43} &= \frac{k_{\text{on}}(R_{\text{tot}} - \hat{x}_4)}{v_3} = \frac{k_{\text{on}} R_{\text{tot}} k_{03}(k_{14} + k_{\text{off}})}{I_{\text{E}} k_{\text{on}} + k_{03} v_3 (k_{14} + k_{\text{off}})}. \end{aligned} \tag{S15}$$

We can also treat the input $u(t)$ as a non-zero initial condition for $x_{1,\text{T}}(t)$.

3 MATHEMATICA CODE FOR GENERATING A_{IJ} AND λ_J

```
(*ODEs for the linear model of tracer kinetics*)
dt1 = -(k21 + k31)*t1[t] + k12*t2[t] + k14*t4[t];
dt2 = k21*t1[t] - k12*t2[t];
dt3 = k31*t1[t] - k03*t3[t] - k43*t3[t] + k34*t4[t];
dt4 = -(k14 + k34)*t4[t] + k43*t3[t];

(*ODEs and initial conditions put into one list to be used by DSolveValue*)
odes = {t1'[t] == dt1, t2'[t] == dt2, t3'[t] == dt3, t4'[t] == dt4};
ics = {t1[0] == d, t2[0] == 0, t3[0] == 0, t4[0] == 0};
eqns = Join[odes, ics];

(*Calculate the A-matrix of the system*)
(aMat = {{-(k21 + k31), k12, 0, k14}, {k21, -k12, 0, 0}, {k31, 0, -k03 -
   k43, k34}, {0, 0, k43, -(k14 + k34)}}) // MatrixForm;

(*\[Lambda]j are given by*)
\[Lambda] = Eigenvalues[aMat];

(*We can look at the expressions for \[Lambda]j in full*)
\[Lambda]Full = Eigenvalues[aMat, Cubics -> True, Quartics -> True];

(*Aij are given by the coefficients of exp(\[Lambda]j*t) in the solution to
   the ODEs*)
sol = DSolveValue[eqns, {t1[t], t2[t], t3[t], t4[t]}, t];

(*Aij can now be found by extracting the coefficients of exp(\[Lambda]j*t)
   in the solution. A14 and A34 are shown here; the others can be computed
   similarly*)
a14 = Coefficient[Normal[sol[[1]]], Exp[t*\[Lambda][[4]]]];
a34 = Coefficient[Normal[sol[[3]]], Exp[t*\[Lambda][[4]]]];

(*We can look at A14 and A34 in full, without Root objects. These
   expressions are extremely long; it is recommended to export them to text
   files*)
a14Full = Replace[a14, {Root[x_, y_] :> ToRadicals[Root[x, y]]}, Infinity];
a34Full = Replace[a34, {Root[x_, y_] :> ToRadicals[Root[x, y]]}, Infinity];
```

4 MATHEMATICA CODE FOR GENERATING RANDOM PARAMETER VECTORS FOR SYNTHETIC DATA STUDY

```
(*literature parameters values*)
litParams = {k21 -> 0.51, k31 -> 0.18, k12 -> 0.41, k14 -> 5.0, k03 -> 3.0,
   koff -> 100., kon -> 1000., v3 -> 0.34, konv3 -> 1000/0.34, Rtot -> 14.,
   v1 -> 2.9, ie -> 15.};

(*expressions for psi1 and psi2*)
\[Psi]1 = (k03*(k14 + koff))/konv3;
\[Psi]2 = k14*Rtot;

(*these are the different sets of parameters we can fix to the literature
   values, in order to estimate the remaining parameters using psi1 and
   psi2*)
rules = {litParams[[{4, 5, 6}]], litParams[[{4, 5, 9}]], litParams[[{5, 6,
   9}]], litParams[[{5, 6, 10}]], litParams[[{5, 9, 10}]], litParams[[{4,
   6, 9}]], litParams[[{6, 9, 10}]]};

(*calculate remaining parameters using psi1 and psi2*)
sol = {Solve[{(\[Psi]1 /. rules[[1]]) == 7.47, (\[Psi]2 /. rules[[1]]) ==
   25.7}, {konv3, Rtot}],Solve[{(\[Psi]1 /. rules[[2]]) == 7.47, (\[Psi]2
   /. rules[[2]]) ==25.7}, {koff, Rtot}],Solve[{(\[Psi]1 /. rules[[3]]) ==
   7.47, (\[Psi]2 /. rules[[3]]) ==25.7}, {k14, Rtot}],Solve[{(\[Psi]1 /.
   rules[[4]]) == 7.47, (\[Psi]2 /. rules[[4]]) ==25.7}, {k14,
   konv3}],Solve[{(\[Psi]1 /. rules[[5]]) == 7.47, (\[Psi]2 /. rules[[5]])
   ==25.7}, {k14, koff}],Solve[{(\[Psi]1 /. rules[[6]]) == 7.47, (\[Psi]2
   /. rules[[6]]) ==25.7}, {k03, Rtot}],Solve[{(\[Psi]1 /. rules[[7]]) ==
   7.47, (\[Psi]2 /. rules[[7]]) ==25.7}, {k03, k14}]};

(*These are the 7 population parameter vectors obtained by setting certain
   parameters to their literature values and calculating the remaining
   parameters so that psi1 and psi2 are equal to their estimated values.
   For each population parameter vector, k21, k12, k31, psi1 and psi2 take
   the same values.*)
popVectors = Table[Join[{k21 -> 0.51, k31 -> 0.154, k12 -> 0.41},
   rules[[i]], sol[[i, 1]], {v1 -> 2.9}], {i, 7}];

(*put them all in the same order*)
pop = {popVectors[[1]], popVectors[[2]][[{1, 2, 3, 4, 5, 7, 6, 8,
   9}]],popVectors[[3]][[{1, 2, 3, 7, 4, 5, 6, 8, 9}]],popVectors[[4]][[{1,
   2, 3, 7, 4, 5, 8, 6, 9}]],popVectors[[5]][[{1, 2, 3, 7, 4, 8, 5, 6,
   9}]],popVectors[[6]][[{1, 2, 3, 4, 7, 5, 6, 8, 9}]],popVectors[[7]][[{1,
   2, 3, 8, 7, 4, 5, 6, 9}]]};

(*verify that parameters estimated from fcr data are consistent*)
```

```
\[Psi]1 /. pop
\[Psi]2 /. pop
k31 /. pop

(*Individual parameter vectors are generated from a lognormal distribution,
   parameterised as follows*)
mu = Table[Log[{k21, k31, k12, k14, k03, koff, konv3, Rtot, v1}] /.
   pop[[p]], {p, 7}];
omega = DiagonalMatrix[Table[0.004, {j, 9}]];

(*number of individuals to simulate*)
n = 41;

(*We wish to simulate 100 uniques sets of 41 individual parameter vectors.
   We also wish to have individuals with a distribution of x1e values
   similar to the real data. Initially we draw more set of 41 individuals
   than we need, then we can select the first 100 of these that meet our
   criteria for x1e*)
lmax = 2000;

(*define distribution for endogenous IgG production rate (ie) separately to
   get an appropriate range of x1e values*)
ieDistribution = MixtureDistribution[{0.8, 0.2},
   {LogNormalDistribution[Log[9.], 1.2], LogNormalDistribution[Log[160.],
   0.25]}];

(*draw random values of ie from the distribution*)
ieValues = Table[Table[Table[RandomVariate[ieDistribution], {j, n}], {l,
   lmax}], {p, 7}];

(*list of 7 (one for each population parameter vector) sets of 2000 sets of
   41 individuals, including endogenosu IgG production rates*)
pvectors =
   Table[Table[Table[Join[RandomVariate[LogMultinormalDistribution[mu[[p]],
   omega]], {ieValues[[p, l, j]]}], {j, n}], {l, lmax}], {p, 7}];

(*We need to check the distribution of x1e is suitable*)
params = Table[Table[Table[{k21 -> pvectors[[p, l, k, 1]], k31 ->
   pvectors[[p, l, k, 2]], k12 -> pvectors[[p, l, k, 3]], k14 ->
   pvectors[[p, l, k, 4]], k03 -> pvectors[[p, l, k, 5]], koff ->
   pvectors[[p, l, k, 6]], konv3 -> pvectors[[p, l, k, 7]],Rtot ->
   pvectors[[p, l, k, 8]], v1 -> pvectors[[p, l, k, 9]], ie -> pvectors[[p,
   l, k, 10]]}, {k, 40}], {l, lmax}], {p, 7}];

(*Calculate x1e for all simulated parameter vectors*)
```

```
x1eExp = (ie (k03 k14 + k03 koff + konv3 ie + k14 konv3 Rtot))/(k31 (k03
   k14 + k03 koff + konv3 ie));
x1e = Table[Table[x1eExp /. params[[p, l]], {l, lmax}], {p, 7}];

(*Find the parameter vectors that have approximately the correct
   proportions of x1e values less than 200, between 200 and 800 and greater
   than 800*)
x1eLe200 = Table[Table[Length[Select[x1e[[p, l]], # < 200. &]]/41., {l,
   lmax}], {p, 7}];
x1e200800 = Table[Table[Length[Select[x1e[[p, l]], # > 200. && # < 800.
   &]]/41., {l, lmax}], {p, 7}];
x1eGe800 = Table[Table[Length[Select[x1e[[p, l]], # > 800. && # < 2000.
   &]]/41., {l, lmax}], {p, 7}];
x1eMax = Table[Table[Max[x1e[[p, l]]], {l, lmax}], {p, 7}];

(*Select the first 100 data sets with the correct distribution of x1e*)
positions =
   Table[DeleteDuplicates[Sort[Flatten[Position[Transpose[{x1eLe200[[p]],
   x1e200800[[p]], x1eGe800[[p]], x1eMax[[p]]}], #] & /@
   Select[Transpose[{x1eLe200[[p]], x1e200800[[p]], x1eGe800[[p]],
   x1eMax[[p]]}], #[[1]] > 0.45 && #[[1]] < 0.55 && #[[2]] > 0.25 && #[[2]]
   < 0.35 && #[[3]] > 0.15 && #[[3]] < 0.25 && #[[4]] > 1500 && #[[4]] <
   2000 &]]]][[1 ;; 100]], {p, 7}];

(*Export 7 datasets (one for each population parameter vector), each
   consisting of 100 set of 41 unique individual parameter vectors*)
Export["params1.mx", pvectors[[1, positions[[1]]]]]
Export["params2.mx", pvectors[[2, positions[[2]]]]]
Export["params3.mx", pvectors[[3, positions[[3]]]]]
Export["params4.mx", pvectors[[4, positions[[4]]]]]
Export["params5.mx", pvectors[[5, positions[[5]]]]]
Export["params6.mx", pvectors[[6, positions[[6]]]]]
Export["params7.mx", pvectors[[7, positions[[7]]]]]
```

5 MATHEMATICA CODE FOR GENERATING SYNTHETIC FCRT DATA

```
(*Import the individual parameter values that were generated for one of the
   population parameter vectors*)
p = Import["params1.mx"];

(*population parameter*)
pop = {k21 -> 0.51`, k31 -> 0.154`, k12 -> 0.41`, k14 -> 5.`, k03 -> 3.`,
   koff -> 100.`, konv3 -> 42.16867469879518`, Rtot -> 5.14`, v1 -> 2.9`,
   ie -> 15.`};

(*Linear model of tracer kinetics*)
```

```
dx1 = -(k21 + k31)*x1[t] + k12*x2[t] + k14*x4[t];
dx2 = k21*x1[t] - k12*x2[t];
dx3 = k31*x1[t] - k03*x3[t] - k43*x3[t] + k34*x4[t];
dx4 = -(k14 + k34)*x4[t] + k43*x3[t];

(*ordinary differential equations*)
odes = {x1'[t] == dx1, x2'[t] == dx2, x3'[t] == dx3, x4'[t] == dx4, x1[0]
   == d\[Mu]mol, x2[0] == 0, x3[0] == 0, x4[0] == 0};

(*solve system ODEs to get solutions for x1(t), x2(t), x3(t), x4(t) *)
x1exp = DSolveValue[odes, x1[t], t];
x2exp = DSolveValue[odes, x2[t], t];
x3exp = DSolveValue[odes, x3[t], t];
x4exp = DSolveValue[odes, x4[t], t];

(*y1(t) and y2(t) are given by*)
y1 = x1exp/d\[Mu]mol;
y2 = (x1exp + x2exp + x3exp + x4exp)/d\[Mu]mol;

(*experimental parameters: dose in micromoles, alpha*)
\[Theta]experiment = {d\[Mu]mol -> 0.005, \[Alpha] -> 45};

(*number of randomly generated cohorts of individuals*)
lmax = 100;
(*number of individual parameter vectors per cohort*)
n = 41;

(*put parameter values in correct format for use*)
params = Table[Table[{k21 -> p[[l, k, 1]], k31 -> p[[l, k, 2]], k12 ->
   p[[l, k, 3]], k14 -> p[[l, k, 4]], k03 -> p[[l, k, 5]], koff -> p[[l, k,
   6]], konv3 -> p[[l, k, 7]], Rtot -> p[[l, k, 8]], v1 -> p[[l, k, 9]], ie
   -> p[[l, k, 10]]}, {k, n}], {l, lmax}];

(*we need to calculate k43 so here is the expression*)
x4hat = konv3*ie*Rtot/(k03*(k14 + k34) + konv3*ie);
k43exp = Simplify[konv3*(Rtot - x4hat)];

(*true parameter vectors including k34 and k43 for simulating linear model*)
\[Theta]inds = Table[Table[Join[params[[l, k]], {k34 -> (koff /. params[[l,
   k]]), k43 -> (k43exp /. (params[[l, k]] /. koff -> k34))},
   \[Theta]experiment], {k, n}], {l, lmax}];
```

```
(*The values of y1 and y2 are calculated from measurements of radioactivity
   concentration in plasma and radioactivity in urine. We therefore define
   the coefficient of variation on these raw measurements. The values were
   set by trial and error*)

(*cv for plasma measurements and dose measurement*)
cv = 0.05
(*cv for urine measurements*)
cvu = 0.05

(*Timepoints for measurements. Assume measurements are taken daily*)
timepoints1 = Table[Table[j, {j, 0, 25}], {k, n}];
timepoints2 = Table[Table[j, {j, 0, 25}], {k, n}];

(*calculate the final timepoints for y1(t) and y2(t)*)
tmax1 = Table[Last[timepoints1[[k]]], {k, n}];
tmax2 = Table[Last[timepoints2[[k]]], {k, n}];
Do[tmax[k] = Max[{tmax1[[k]], tmax2[[k]]}], {k, n}]

(*radioactivity concentration in plasma, i.e. measured variable from which
   we calculate y1(t) *)
plasmaRadConc = x1exp*\[Alpha]/v1;

(*calculate the true values of plasmaRadConc at all timepoints to speed up
   next step*)
plasmaRadConcPts = Table[Table[Re[plasmaRadConc /. \[Theta]inds[[l, k]] /.
   t -> timepoints1[[k]]], {k, n}], {l, lmax}];

(*For our n individuals for each cohort, generate a dataset for
   plasmaRadConc [uCi/ml], assuming that this measurement has constant cv *)
plasmaRadConcTable = Table[Table[Table[plasmaRadConcPts[[l, k, j]] +
   RandomVariate[NormalDistribution[0, cv*plasmaRadConcPts[[l, k, j]]]],
   {j, Length@plasmaRadConcPts[[l, k]]}], {k, n}], {l, lmax}];

(*calculate y1(t) measurements*)
y1Table = Table[Table[plasmaRadConcTable[[l, j]]/plasmaRadConcTable[[l, j,
   1]], {j,n}], {l, lmax}];

(*The radiation in urine is given by*)
radUrine = Simplify[\[Alpha]*(d\[Mu]mol - x1exp - x2exp - x3exp - x4exp)];

(*Calculuate the radioactivity in urine collections with a constant CV
   (proportional error model)*)
```

```
dailyRadUrineTable = Table[Table[Table[dailyRadUrine[[l, k, j]] +
   RandomVariate[NormalDistribution[0, cvu*dailyRadUrine[[l, k, j]]]], {j,
   Length@dailyRadUrine[[l, k]]}], {k, n}], {l, lmax}];

(*The calculated cumulative urine radioactivity is given by*)
radUrineTable = Table[Table[Prepend[Accumulate[dailyRadUrineTable[[l, k]]],
   0], {k, n}], {l, lmax}];

(*the radioactivity in the dose is given by radDose = alpha*d\[Mu]mol*)
radDose = \[Alpha]*d\[Mu]mol;

(*Simulate measurements of the radioactivity measured in the tracer \
dose for the 40 subjects and repeat lmax times*)
radDoseTable = Table[Table[(radDose /. \[Theta]experiment) +
   RandomVariate[NormalDistribution[0, cv*(radDose /.
   \[Theta]experiment)]], {k, n}], {l, lmax}];

(*Calculate y2(t) *)
y2Table = Table[Table[1 - (radUrineTable[[l, k]]/radDoseTable[[l, k]]), {k,
   n}],{l,
lmax}];

(*plot one example of noisy measurements of y1(t)*)
y1plot = Show[ListPlot[Table[Transpose[{timepoints1[[k]], y1Table[[1,
   k]]}], {k, n}], Joined -> False, PlotRange -> All],
   Plot[Evaluate[Table[y1 /. \[Theta]inds[[1, k]], {k, n}]], {t, 0, 25},
   PlotRange -> All]]

(*plot one example of noisy measurements of y2(t)*)
y2plot = Show[ListPlot[Table[Transpose[{timepoints2[[k]], y2Table[[1,
   k]]}], {k, n}]], Plot[Evaluate[Table[y2 /. \[Theta]inds[[1, k]], {k,
   n}]], {t, 0, 25}, PlotRange -> All]]


(*calculate the FCR for each subject as if it is measured every day*)
fcrDaily = Table[Table[((dailyRadUrineTable[[l, k]]/radDoseTable[[l,
   k]]))/(Drop[y1Table[[l, k]], 1]), {k, n}], {l, lmax}];

(*We assume that the experimenter takes an average value for the latter
   part of the experiment. We therefore calculate the mean of the last 5
   FCR measurements for each subject*)
measuredFCR = Table[Table[Mean[fcrDaily[[l, k, 16 ;; 25]]], {k, n}], {l,
   lmax}];

(* calculate true x1e *)
```

```
x1eExp = (ie (k03 k14 + k03 koff + konv3 ie + k14 konv3 Rtot))/(k31 (k03
   k14 + k03 koff + konv3 ie));
x1e = Table[x1eExp /. params[[l]], {l, lmax}];

(*plot measured FCR for the first 10 sets of subjects*)
Table[Show[ListPlot[Transpose[{x1e[[l]], measuredFCR[[l]]}], PlotRange ->
   All],Plot[fcrE /. pop, {q1, 0, 2000}, PlotStyle -> ColorData[97, 2],
   PlotRange -> All], AspectRatio -> 1, PlotRange -> {{0, 2000}, All}], {l,
   10}]

(*Put the FCRT data in a form that we can export for parameter estimation*)
fcrData = Table[Transpose[{x1e[[l]], measuredFCR[[l]]}], {l, lmax}];
```
